# Isotropic shrinkage of patterned vacancies enables three-dimensional nanoprecise metastructures for visible light applications

**DOI:** 10.1038/s41566-026-01896-1

**Published:** 2026-05-12

**Authors:** Quansan Yang, Gaojie Yang, Takahiro Nambara, Hiroyuki Kusaka, Yuichiro Kunai, Alex C. Matlock, Corban Swain, Brett Pryor, Yannick Salamin, Daniel Oran, Hasindu Kariyawasam, Ramith Hettiarachchi, Dushan Wadduwage, Marin Soljačić, Peter T. C. So, Edward S. Boyden

**Affiliations:** 1 McGovern Institute for Brain Research, Massachusetts Institute of Technology (MIT), Cambridge, MA USA; 2 Department of Mechanical Engineering, MIT, Cambridge, MA USA; 3 Department of Materials Science and Engineering, University of Washington, Seattle, WA USA; 4 Fujikura Ltd., Tokyo, Japan; 5 Department of Physics, MIT, Cambridge, MA USA; 6 Research Laboratory of Electronics, MIT, Cambridge, MA USA; 7 CREOL, The College of Optics and Photonics, University of Central Florida, Orlando, FL USA; 8 MIT Media Lab, MIT, Cambridge, MA USA; 9 Irradiant Technologies Inc., Waltham, MA USA; 10 Center for Advanced Imaging, Harvard University, Cambridge, MA USA; 11 Department of Computer Science, Old Dominion University, Norfolk, VA USA; 12 School of Data Science, Old Dominion University, Norfolk, VA USA; 13 Department of Physics, Old Dominion University, Norfolk, VA USA; 14 Department of Biological Engineering, MIT, Cambridge, MA USA; 15 Department of Brain and Cognitive Sciences, MIT, Cambridge, MA USA; 16 Howard Hughes Medical Institute, MIT, Cambridge, MA USA; 17 K. Lisa Yang Center for Bionics and Yang Tan Collective, MIT, Cambridge, MA USA; 18 Center for Neurobiological Engineering, MIT, Cambridge, MA USA; 19 Koch Institute for Integrative Cancer Research, MIT, Cambridge, MA USA

**Keywords:** Materials for optics, Nanoscience and technology, Optical materials and structures

## Abstract

Three-dimensional metastructures with nanoscale feature sizes exhibit unique properties compared with structures with larger feature sizes, but are difficult to fabricate. Here we introduce implosion carving (ImpCarv), a method for photopatterning vacancies of complex geometry throughout materials, followed by isotropic shrinkage (>10-fold). ImpCarv works by photoactivating sensitizers to generate reactive oxygen species that cleave a swollen hydrogel at defined points, followed by controlled shrinkage via dehydration. ImpCarv creates three-dimensional metastructures where the refractive index of each point throughout a material can be specified with nanoscale precision via material presence or absence. By leveraging refractive index programmability for precise phase control, we demonstrate an all-optical machine learning device with nanoscale neuron sizes operating at visible wavelengths. ImpCarv may thus support diverse applications in nanophotonics and nanotechnology.

## Abstract (web summary)

A method that combines photopatterning with 10-fold shrinkage can fabricate three-dimensional metastructures with nanoscale features for applications with visible light.

## Main

Three-dimensional (3D) metastructures with nanoscale feature sizes are much desired in fields such as photonics^1–4^. Additive manufacturing approaches^5–9^, such as multi-photon polymerization and more advanced, stimulated-emission-depletion-inspired lithography, have been used to create such structures, with mechanically self-supporting geometries, but complex geometries (for example, arbitrary-shaped internal voids and extreme aspect ratios) remain challenging^10–12^. Subtractive manufacturing approaches, which cleave scaffolds at photo-targeted points to create vacancy patterns throughout materials, could enable such geometries^13–16^, but so far have exhibited feature sizes diffraction limited by the wavelength of the applied light (typically in the many hundreds of nanometres)^17^. If we could photopattern vacancies in 3D throughout a material, with resolution in the tens of nanometres (far smaller than the wavelength of applied light), the resulting refractive index distributions could support nanophotonic control of visible light across a wide array of optical applications^18–20^. We here extend the concept of implosion fabrication^21^ towards the creation of such vacancies, to enable nanoprecise refractive index control throughout 3D metastructures.

In implosion fabrication and similar methods, a hydrogel or other scaffold material, equipped with specific materials at photo-targeted (or otherwise specified) sites, is shrunk to achieve a final configuration of the deposited materials with nanoscale (or greater) feature sizes^4,21,22^. Increasing the refractive index at defined points throughout a hydrogel through deposition of nanoparticles would in principle be possible^21,23^, but achieving the desired optical properties of the nanoparticles, while retaining controlled and predictable surface chemistry to avoid non-specific binding or aggregation during hydrogel infusion, is challenging^24,25^. This complexity provided us with motivation to explore whether nanoprecise vacancy creation could achieve such refractive index control without the need for custom materials.

Here we present implosion carving (ImpCarv), a method for fabrication of 3D metastructures with resolution down to tens of nanometres by creating vacancies throughout a hydrogel scaffold via photopatterning using conventional diffraction-limited optics, followed by isotropic scaffold shrinkage via dehydration. ImpCarv proceeds through three steps: first, we perform two-photon activation of sensitizers to cleave a swollen hydrogel at defined sites to create vacancies. These sensitizers absorb light and generate reactive oxygen species (ROS)^26^, which enable targeted cleavage of the hydrogel scaffold^27^, given the limited ROS diffusion range in aqueous environments (~100 nm)^28^. Second, we shrink the hydrogel ~10-fold linearly through adding divalent cations^4,21^ so that features are brought into the resolution range of tens of nanometres. Third, supercritical drying is used to preserve vacancies during scaffold dehydration^29^, resulting in additional shrinkage. The net outcome is a high refractive index contrast of ~0.5 between vacancies and neighbouring scaffold material, enabling 3D nanoprecise control of the refractive index throughout metastructures. With this refractive index programmability, our devices can achieve precise phase control of light.

One use of such devices is in optical computing, here exemplified by diffractive optical networks, which leverage 3D refractive index distributions within structures to perform machine learning tasks, enabling information processing at the speed of light, via passive operation^30^. These devices have proven effective for various applications, including image classification^31^, object and pattern recognition^32^, signal processing^33^ and computational imaging^34^. However, they either operate in long-wavelength regimes (infrared and longer) or require large machine learning unit (that is, neuron) sizes, which require centimetre-scale axial dimensions owing to constraints in the diffraction angles of the resulting neurons^35^. Ideally, one would be able to handle visible light inputs, and utilize nanoscale neuron sizes, to facilitate miniaturization and integration^36^. Here we present an all-optical machine learning device with nanoscale neuron sizes capable of operating at visible wavelengths. We show its usefulness in a digit classification task. Such a nanophotonic device could open up new frontiers in machine learning-based optical computing and highlights the power of ImpCarv in nanophotonics.

## Implosion carving workflow and initial validation

**Fig. 1:**
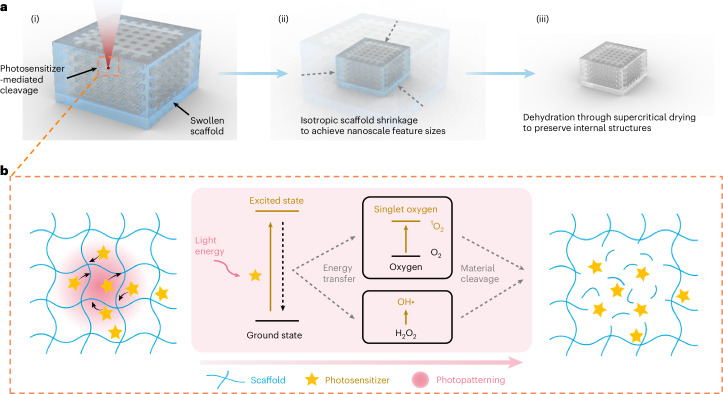
Implosion carving workflow and initial validation. **a**, Schematic illustration of the ImpCarv process: (i) 3D photosensitizer-mediated cleavage within the scaffold, (ii) isotropic scaffold shrinkage to achieve nanoscale feature sizes and (iii) dehydration through supercritical drying to preserve internal structures. **b**, Mechanism of photosensitizer-mediated cleavage (not to scale). Photopatterning activates photosensitizers from their ground state to an excited state, undergoing photochemical reactions to generate ROS, including ^1^O_2_ and HO·, leading to the cleavage of hydrogel backbone chains. Schematics in **b** created in BioRender; Yang, Q. https://biorender.com/2oqeko9 (2026).

Swollen polyacrylate hydrogels were selected as scaffolds for ImpCarv owing to their known ability to shrink by approximately 10-fold in linear dimension upon dehydration^4,21^. We used throughout this paper (unless otherwise stated) two distinct polyacrylate hydrogel formulations with varying cross-linker concentration to achieve different final shrinkage factors: a high-shrinkage-factor (HSF) hydrogel (0.026 wt% cross-linker; detailed formulation in Supplementary Table 1) and a moderate-shrinkage-factor (MSF) hydrogel (0.240 wt% cross-linker; detailed formulation in Supplementary Table 2), which can achieve final shrinkage factors of approximately 13 and 5, respectively, after dehydration. To induce vacancies throughout these hydrogels, we drew inspiration from photodynamic therapy, a technique using photosensitizers to selectively ablate cancer cells, pathogenic microbes and unwanted tissues^37^. Photosensitizers, activated by light, undergo photochemical reactions to generate ROS, for targeted destruction of cells^26,37^. Following this principle, we developed a process involving immersion of hydrogel scaffolds in an aqueous photosensitizer (that is, rhodamine B) solution (throughout the ImpCarv process, this solution, along with all other solutions used, was applied in large excess relative to the hydrogel volume), followed by multi-photon laser exposure to activate rhodamine B from its ground state to its excited state in 3D photo-targeted regions. This activation generates ROS, including singlet oxygen (^1^O_2_) and hydroxyl radicals (·OH), with a limited diffusion distance (~100 nm) from the laser excitation point^28^, leading, we found, to the cleavage of the hydrogel backbone chains (schematized in Fig. 1a(i),b).

Fluorescence imaging indicated that laser power too weak to achieve cleavage caused rhodamine B to bind to the hydrogel backbone (as in original implosion fabrication^21^), resulting in higher fluorescence intensity in these regions compared with surrounding non-patterned regions; as laser power increased, material cleavage started in the photopatterned regions, leading to a reduction in fluorescence intensity (Supplementary Fig. 1a,b). Around material cleavage sites, some rhodamine B remained anchored at the boundaries of the photopatterned regions, likely owing to the insufficient laser power at the periphery of the laser focal spot (bottom right inset in Supplementary Fig. 1b). In subsequent experiments, rhodamine B at the boundary of a cleaved region served as a marker to characterize structures post-cleavage through fluorescence imaging. In addition, consistent with findings from photodynamic therapy^38^, we observed that adding oxygen (for example, by bubbling the aqueous rhodamine B solution with oxygen (O_2_)) and hydrogen peroxide (H_2_O_2_) enhanced cleavage efficiency by lowering the required laser power (Supplementary Fig. 1b–d). These assays utilized fluorescence as an indirect way of characterizing the vacancy; below, after we talk through the shrinkage process, we will discuss electron microscopy validation. To maintain the hydrogels in their swollen state during photopatterning, isopropylamine was added to the aqueous photosensitizer solution, causing rhodamine B-induced hydrogel shrinkage to go from ~50% reduction in the hydrogel size to ~0% reduction, likely owing to acid–base complexation between hydrogel carboxyl groups and isopropylamine^39^, with a resulting solution pH of approximately 8.0–9.5. After photopatterning, to prevent the ~20% shrinkage caused by washing in pure water, we washed hydrogels in an aqueous isopropylamine solution of the same pH as the photosensitizer solution for microscopy characterization of the patterned hydrogel before controlled shrinkage.

We designed a series of procedures to induce scaffold shrinkage using divalent and monovalent cations, because these hydrogel scaffolds are negatively charged polyelectrolytes^21,40^ (schematized in Fig. 1a(ii)). To prevent the hydrogel cracks that we saw when divalent cations (for example, magnesium chloride (MgCl_2_)) were applied immediately to swollen hydrogels, we first immersed them in a solution of monovalent cations (that is, sodium chloride (NaCl), 0.02 M in pure water, 15 min), followed by immersion in MgCl_2_ (0.1, 0.3 and 0.5 M in pure water, 30 min each), resulting in a final shrinkage factor of 4.78 ± 0.04 for HSF hydrogels (mean ± standard deviation (s.d.) used throughout, *n* = 7 areas from 3 gels) and 3.28 ± 0.25 for MSF hydrogels (*n* = 5 areas from 5 gels) (we will call this sodium–magnesium treatment for short; full procedure flowchart in Supplementary Fig. 2). Further increases in MgCl_2_ concentration (for example, to 1.0 M) did not result in additional noticeable shrinkage. Calcium cations exhibit higher binding affinity to carboxylic acid groups compared with magnesium cations^41^. Following the treatment with 0.5 M MgCl_2_, we immersed the hydrogels in calcium chloride (CaCl_2_, 0.1 M, 0.3 M and 0.5 M in pure water, 30 min each), to achieve a final shrinkage factor of 9.47 ± 0.15 for HSF hydrogels (*n* = 4 areas from 2 gels) and 3.78 ± 0.06 for MSF hydrogels (*n* = 7 areas from 7 gels) (we will call this calcium treatment for short; Supplementary Fig. 2). Increasing the CaCl_2_ concentration beyond this range (for example, to 5.0 M) did not yield further noticeable shrinkage. For certain experiments (see the final paragraph of this section), owing to the limited resolution of fluorescence imaging, we only immersed the swollen hydrogels in NaCl (1.0 M in pure water) to induce a modest shrinkage factor of approximately 2 for HSF hydrogels, so that structural features could be preserved at the microscale for fluorescence imaging.

The third step involved dehydrating the hydrogel scaffold via supercritical drying to preserve internal structures (schematized in Fig. 1a(iii)). Initial attempts at ambient air drying resulted in the collapse of these structures, presumably owing to surface tension and capillary forces^42^, as evidenced by an obvious reduction in intensity contrast between the internal structures and the hydrogel scaffold under an optical microscope. We also tested freeze-drying; however, this method caused the hydrogel to become opaque, consistent with previous findings^43^, possibly owing to light scattering from microscale pores formed during the process^44^. Inspired by microelectromechanical system fabrication, we utilized supercritical drying to dehydrate these structures while minimizing surface tension and capillary forces^29^. After the treatment with 0.5 M CaCl_2_, to prevent the occurrence of hydrogel opaqueness that we saw caused by solvent exchange from 0.5 M CaCl_2_ to ethanol (the solvent required for supercritical drying), likely owing to the phase separation of hydrogel backbone chains^45^, we gradually increased the CaCl_2_ concentration from 0.5 M to 5.0 M in pure water by slowly infusing 5.0 M CaCl_2_ while keeping the total volume constant (infusion and removal rates, 0.25 ml h^−1^; duration, 48 h; initial solution volume, ~5 ml; estimated CaCl_2_ concentration after infusion and removal, ~4.6 M), followed by immersing the hydrogels in 4.8 M and 5.0 M CaCl_2_ for 30 min each. Subsequently, we replaced the 5.0 M CaCl_2_ with ethanol by gradually increasing the ethanol concentration from 0 vol% to 100 vol% in 5 vol% increments within the 5.0 M CaCl_2_–ethanol mixture (30 min each) (we will call this ethanol solvent exchange for short). The ethanol solvent exchange led to a further increase in the shrinkage factor (11.71 ± 0.58 for HSF hydrogels, *n* = 7 areas from 3 gels; 4.17 ± 0.01 for MSF hydrogels, *n* = 3 areas from 3 gels; Supplementary Fig. 2), possibly owing to changes in solvent–scaffold interactions associated with reduced solvent polarity^46^. Supercritical drying was then used, using a commercial critical point dryer with default parameters, to replace ethanol with liquid carbon dioxide (CO_2_), and to reach the CO_2_ critical point to minimize surface tension and capillary forces, followed by the removal of CO_2_ (ref. ^47^). After supercritical drying, the scaffold remained transparent, and the internal structures exhibited a marked intensity contrast relative to the scaffold under an optical microscope (Supplementary Fig. 3). Notably, the supercritical drying process resulted in a final shrinkage factor of 13.18 ± 0.28 for HSF hydrogels (*n* = 7 areas from 4 gels) and 5.03 ± 0.06 for MSF hydrogels (*n* = 5 areas from 5 gels) (Supplementary Fig. 2), presumably owing to the changes in solvent–scaffold interactions as ethanol was replaced by liquid CO_2_ (ref. ^47^). Detailed fabrication procedures are provided in Methods.

**Fig. 2:**
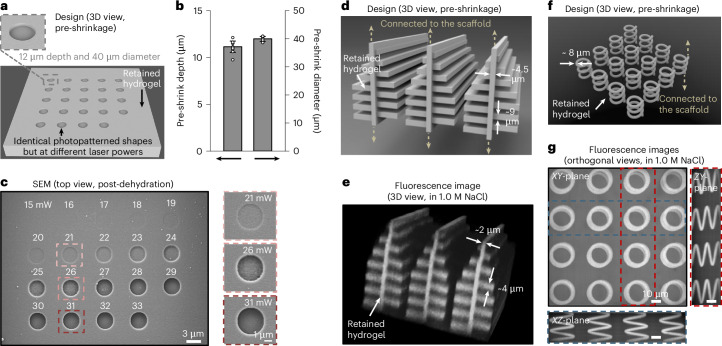
Validation and complex geometries. **a**, Design of an array of circular holes with identical dimensions within the hydrogel scaffold. Inset: designed dimensions of circular holes with a depth of 12 µm and a diameter of 40 µm. **b**, Measured dimensions of the structures described in **a** treated with laser powers of 28 mW or greater in swollen hydrogel: depth of 11.2 ± 0.6 µm and diameter of 40.0 ± 0.9 µm (mean ± s.d. used throughout, *n* = 12 circular holes from 2 arrays from 2 gels; open circular points, bars and error bars represent individual data points, means and s.d., respectively, used throughout; HSF hydrogels were used throughout this figure). **c**, SEM images (from one gel) of the structure described in **a** post-dehydration (surface-coated with 10-nm-thick Pd/Pt). Insets: magnified SEM images of circular holes created with laser powers of 21 mW (top right), 26 mW (middle right) and 31 mW (bottom right). **d**, Design of a high-aspect-ratio structure resembling a butterfly wing within the hydrogel, with both top and bottom ends connected to the hydrogel scaffold. **e**, Representative maximum-intensity projection of *z*-stacked fluorescence images (*n* = 4 structures from 3 gels) of the structure described in **d** after approximately 2-fold shrinkage in 1.0 M NaCl. **f**, Design of a 3D helical array within the scaffold, with both top and bottom ends connected to the hydrogel scaffold. **g**, Representative maximum-intensity projection of *z*-stacked fluorescence images (*n* = 4 structures from 3 gels) in the *XY*-plane of the structure from **f** after approximately 2-fold shrinkage in 1.0 M NaCl. Blue inset: maximum-intensity projection of the fluorescence images in the *XZ*-plane corresponding to the blue dashed frame in **g**. Red inset: maximum-intensity projection of the fluorescence images in the *ZY*-plane corresponding to the red dashed frame in **g**. Source data

To evaluate the photopatterned structures, we designed an array of circular holes with identical dimensions (depth, 12 µm; diameter, 40 µm) within the hydrogel scaffold (schematized in Fig. 2a). These circular holes were patterned inside the hydrogel at different laser powers (ranging from 15 mW to 33 mW); subsequently, the hydrogel above these circular holes was removed using a higher laser power (that is, 40 mW) to expose the surface for scanning electron microscopy (SEM). The single *z*-slice fluorescence image of this structure in swollen hydrogel indicated complete material cleavage in the photopatterned regions above a certain laser power threshold (~28 mW for HSF hydrogels) (Supplementary Fig. 4a). Fluorescence imaging revealed that the measured dimensions of the photopatterned regions with complete material cleavage treated with laser powers of 28 mW or greater were 11.2 ± 0.6 µm in depth and 40.0 ± 0.9 µm in diameter (*n* = 12 circular holes from 2 arrays from 2 gels) (Fig. 2b and Supplementary Fig. 4b–f), which corresponds to the designed dimensions. Post-dehydration, SEM imaging (surface-coated with 10-nm-thick palladium/platinum (Pd/Pt)) demonstrated that laser powers of 28 mW or greater achieved material cleavage with the target shape and a smooth surface (Fig. 2c and bottom right inset using 31 mW as an example). By contrast, subthreshold laser powers resulted in incomplete material cleavage (top right inset in Fig. 2c using 21 mW as an example) and/or a rough surface (middle right inset in Fig. 2c using 26 mW as an example). Additional atomic force microscopy (AFM) measurements indicated that the root-mean-square roughness of the surface of the photopatterned regions with a laser power above threshold (that is, 30 mW) post-dehydration was 6.3 ± 1.4 nm (*n* = 3 areas from 3 gels) (Supplementary Fig. 5). This roughness was much smaller than the wavelength of light divided by the implosion factor, likely because of a known effect—namely that adjacent voxels during photopatterning exhibited lateral displacements narrower than the focal width of the laser beam, and thus resulted in a smoothing effect (for example, each point on the surface was effectively visited by the laser more than once, causing smoothing)^48^.

To evaluate whether our method is compatible with complex geometries, we fabricated two representative examples that are challenging to produce using additive manufacturing approaches. The first example was a high-aspect-ratio structure resembling nanostructures within a *Morpho* butterfly wing^49^ (schematized in Fig. 2d). The fabrication of such structures has not been achieved so far using additive manufacturing approaches, primarily owing to the high risk of structural collapse when producing high-aspect-ratio features^11^. Leveraging the subtractive manufacturing of ImpCarv, we designed such a structure (with top and bottom ends connected to the hydrogel scaffold, not shown in the schematic). After photopatterning vacancy around the *Morpho* structure, the retained non-patterned hydrogel exhibited an aspect ratio of approximately 30 (central pillar height and width were ~60 µm and ~2 µm, respectively, after a modest shrinkage factor of ~2 in 1.0 M NaCl, to facilitate confocal imaging; Fig. 2e). A second example was a 3D helical structure (schematized in Fig. 2f). While 3D helical microstructures can be fabricated using additive manufacturing approaches, these approaches required a tapering pedestal that gradually evolves into the thin thread of the helix, to prevent the thin thread from collapsing^12^. By contrast, the subtractive manufacturing approach in ImpCarv enabled us to make a helix with a constant diameter thread, simply by attaching the top and bottom ends of the thread to the hydrogel scaffold, without requiring a broadening to a more supportive base. After photopatterning vacancy around the helices, helices were able to freely stand as such (again with a shrinkage factor of ~2 in 1.0 M NaCl), as illustrated in the *XY*-plane (maximum-intensity projection of *z*-stacked fluorescence images; Fig. 2g), with the *XZ*-plane shown in the blue inset (maximum-intensity projection of the same fluorescence images, corresponding to the blue dashed frame in the *XY*-plane) and the *ZY*-plane shown in the red inset (maximum-intensity projection of the same fluorescence images, corresponding to the red dashed frame in the *XY*-plane).

## Isotropic shrinkage of photopatterned vacancies enables nanoscale resolution

**Fig. 3:**
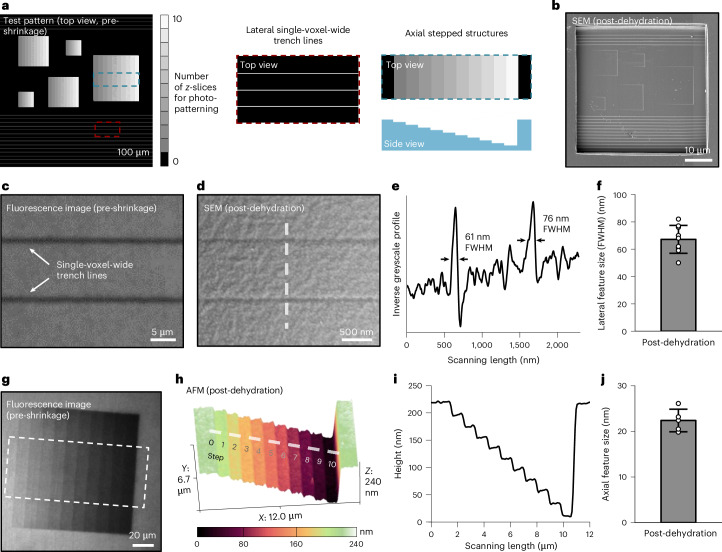
Implosion yields nanoscale resolution vacancies. **a**, Design of the test pattern, including lateral single-voxel-wide trench lines (detailed in the red inset) and axial stepped structures (detailed in the blue inset and in the schematic of the cross-sectional height profile at the bottom right). The fabrication of the axial stepped structures, which contained 11 steps, was achieved by photopatterning 10 *z*-slices (step size between consecutive slices, 250 nm), with each slice ablating an additional portion of the polymer. **b**, SEM image (*n* = 2 test patterns from 2 gels) of the test pattern described in **a** post-dehydration (surface-coated with 10-nm-thick Pd/Pt; HSF hydrogels were used throughout this figure). The pattern was initially generated within the scaffold, and the hydrogel above the pattern was cleaved to expose the surface for imaging. **c**, Representative single *z*-slice fluorescence image (*n* = 25 lines from 2 gels) of 2 lateral single-voxel-wide trench lines in swollen hydrogel. **d**, Representative SEM image (*n* = 7 lines from 3 gels) of the structure described in **c** post-dehydration (surface-coated with 10-nm-thick Pd/Pt). **e**, Cross-sectional inverse greyscale profile of the two single-voxel-wide trench lines along the dashed line direction in **d**, with their FWHM values calculated according to Gaussian single peak fitting. **f**, FWHM values of these single-voxel-wide trench lines are 67 ± 12 nm (mean ± s.d.; *n* = 7 lines from 3 gels). **g**, Representative sum-intensity projection of *z*-stacked fluorescence images (*n* = 2 stepped structures from 2 gels) of the axial stepped structure in a swollen hydrogel. **h**, Representative AFM image (*n* = 5 stepped structures from 2 gels) of the structure from the white dashed frame in **g** post-dehydration. **i**, Cross-sectional profile, along the white dashed line direction in **h**, indicating an average step height difference of approximately 20 nm across the structure. **j**, Statistical step height values are 22 ± 2 nm (mean ± s.d.; *n* = 5 stepped structures from 2 gels). Source data

We anticipated that the minimum feature size of ImpCarv would be nanoscale, as in implosion fabrication^21^. To validate this, we designed a test pattern (schematized in Fig. 3a) comprising lateral single-voxel-wide trench lines (red inset of Fig. 3a; where the width of a single voxel corresponds to the focal width of the laser in our multi-photon system) and axial stepped structures (blue inset, and schematic of cross-sectional height profile at the bottom right, of Fig. 3a). The fabrication of the axial stepped structures, which contained 11 steps, was achieved by photopatterning 10 *z*-slices (step size between consecutive slices, 250 nm), with each slice retaining a different fraction of the polymer. In other words, the resulting axial dimension of a given step was determined by the number of *z*-slices at that step for which the pattern specified removal of the polymer. We now did full shrinkages through the multi-stage method described above, to probe ultimate resolution, and thus used SEM and AFM to characterize the nanoscale nature of these structures. The test pattern was generated within a hydrogel scaffold, followed by cleavage of the hydrogel above the design pattern to expose the surface for imaging (a representative SEM image is shown in Fig. 3b).

We first examined the lateral single-voxel-wide trench lines in swollen hydrogels through single *z*-slice fluorescence imaging (Fig. 3c) and then characterized the corresponding structures post-dehydration through SEM imaging, facilitated by coating the samples with a 10-nm-thick Pd/Pt layer (Fig. 3d). The full-width at half-maximum (FWHM) values of these 2 single-voxel-wide trench lines, along the white dashed line in Fig. 3d, were found to be 61 nm and 76 nm, respectively (Fig. 3e; population data 67 ± 12 nm (*n* = 7 lines from 3 gels) in Fig. 3f). In this study, we used a 20× objective with a numerical aperture (NA) of 1.00 for photopatterning, and there is potential to achieve even more precise structures through the use of objectives with higher NAs.

We evaluated the axially stepped structures in swollen hydrogels through a sum-intensity projection of *z*-stacked fluorescence images (Fig. 3g), and subsequently characterized the structures post-dehydration through AFM imaging (Fig. 3h, corresponding to the white dashed frame in Fig. 3g). AFM measurements indicated an average step height difference of approximately 20 nm across the structure (Fig. 3i, cross-sectional profile along the white dashed line in Fig. 3h), averaging 22 ± 2 nm (*n* = 5 stepped structures from 2 gels) (Fig. 3j). More precise axial feature sizes could be achieved, in principle, with enhanced axial control in photopatterning by using advanced stepper motors. These SEM and AFM measurements confirmed that ImpCarv achieved resolutions down to the tens of nanometres, both laterally and axially.

The proposed photosensitizer-mediated cleavage strategy, the first key step of ImpCarv, is compatible with a wide range of hydrogels, beyond the polyacrylate hydrogels mentioned above. We examined several widely used hydrogels, including agarose, alginate and gelatin, using rhodamine B as the photosensitizer, and found that photopatterned regions were dark, and flanked by fluorophore-bearing neighbourhoods as before (Supplementary Fig. 6). Our method is also compatible with a wide range of photosensitizers (Supplementary Fig. 7). Achieving final nanoprecision, however, requires that the material also supports isotropic shrinkage and supercritical drying, which needs to be individually optimized for each material system, as we have done here for poly(acrylate-co-acrylamide) scaffolds.

## Refractive index programmability and precise phase control

**Fig. 4:**
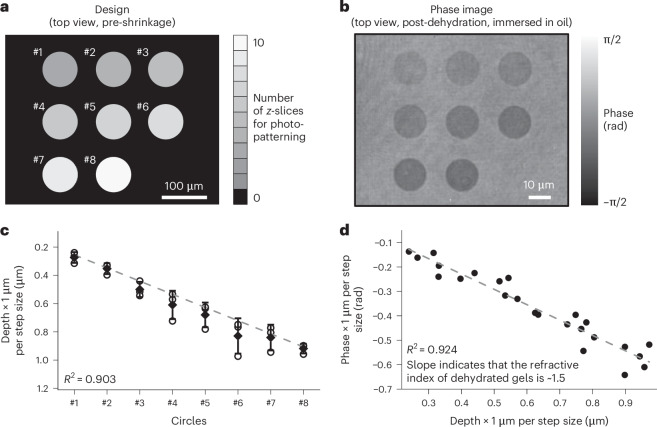
Refractive index programmability and precise phase control. **a**, Design of the test pattern, consisting of eight circles with different depths (#1–8). The fabrication of these circles, with varied depths, was achieved by photopatterning 10 *z*-slices (the circle #*n* has *n* + 2 *z*-slices; step size between consecutive slices, 1.0 µm and 0.75 µm, each for one gel), with each slice ablating an additional portion of the polymer. **b**, Representative phase image (*n* = 3 test patterns from 2 gels; the 2 gels are identical except for the step sizes) of the structure described in **a** post-dehydration (MSF hydrogels were used throughout this figure), observed at a wavelength of 532 nm. The gels were immersed in oil (refractive index, 1.48) for phase imaging with low noise. **c**, Statistical analysis of depth values ($h$) for these circles described in **b** (*n* = 3 test patterns from 2 gels; the 2 gels are identical except for the step sizes; open circular points, solid rhombic points and error bars represent individual data points, means and s.d., respectively). These depth values were measured by AFM, and then for the gel with a step size of 0.75 µm, the depth values were linearly converted to values assuming a step size of 1.0 µm for better comparison, using the equation ${h}_{\mathrm{converted}}={h}_{\mathrm{original}}\times \,1\,{\upmu {\mathrm{m}}}/0.75\,{\upmu {\mathrm{m}}}$. The grey dashed line refers to linear regression of the 24 data points in total performed to assess the relationship between the depth values and the circle number, with a coefficient of determination of 0.903 (*R*^2^). The *t*-test for the slope coefficient (*t*(22) = 14.29, *P* = 1.3 × 10^−12^) indicates a statistically significant linear relationship between the depth values and circle numbers. **d**, Statistical analysis of phase values ($\varphi$) as a function of depth values for these circles described in **b** (*n* = 3 test patterns from 2 gels; the 2 gels are identical except for the step sizes; solid circular points represent individual data points). For the gel with a step size of 0.75 µm, the phase and depth values were linearly converted to values assuming a step size of 1.0 µm for better comparison, using the equation ${\varphi }_{\mathrm{converted}}={\varphi }_{\mathrm{original}}\,\times \,1\,{\upmu {\mathrm{m}}}/0.75\,{\upmu {\mathrm{m}}}$ for $\varphi$ and the equation in **c** for $h$. The grey dashed line refers to linear regression performed to assess the relationship between the phase and the depth values, with a coefficient of determination of 0.924 (*R*^2^). The *t*-test for the slope coefficient (*t*(22) = −16.36, *P* = 8.5 × 10^−14^) indicates a statistically significant linear relationship between the phase and depth values. The slope yields a refractive index of ~1.5 for the post-dehydrated gels, based on the equation $\Delta \varphi =(2\pi /\lambda )\times \Delta n\times \Delta h$, where $\lambda$ is the wavelength (532 nm). Source data

We next characterized the refractive index contrasts yielded by the ImpCarv process ($\Delta n$; the difference in refractive indices between photopatterned and non-patterned regions) and the resulting phase changes enabled by these refractive index contrasts ($\Delta \varphi$; the difference in phase between photopatterned and non-patterned regions; relationship between $\Delta \varphi$ and $\Delta n$,

1 $$\Delta \varphi =(2\pi \times \Delta n\times h)/\lambda$$

where $h$ is the depth of the photopatterned regions and $\lambda$ is the wavelength). The observed $\Delta n$ for HSF hydrogels after sodium–magnesium treatment (5-fold shrinkage) was approximately 0.026 between photopatterned vacancies (containing water) and non-patterned regions, as characterized by diffraction phase microscopy (Supplementary Fig. 8). After further dehydration through supercritical drying, vacancies would fill with air and thus exhibit even higher phase contrast^50^. After dehydration (test pattern, Fig. 4a; phase image, Fig. 4b) via supercritical drying, precise depth control was achievable (Fig. 4c). The slope of the phase versus depth yields a refractive index of ~1.5 for the post-dehydrated gels, resulting in a $\Delta n$ of ~0.5 between the post-dehydrated gels and the air-filled vacancies. We further validated the refractive index of post-dehydrated gels using an index-matching method^51^, which also indicated a value of ~1.5 (Supplementary Fig. 9).

With the refractive index programmability and precise phase control, to explore the potential of ImpCarv for nanophotonics, we created two start-of-the-art nanoprecise 3D structures^8,9^. Supplementary Fig. 10 shows nanoprecise 3D woodpile photonic crystals fabricated with ImpCarv that exhibit strong structural colours, indicating a photonic bandgap in the visible spectral range. Supplementary Fig. 11 presents nanoprecise 3D vacant spirals fabricated with ImpCarv, with a diameter of ~2 µm and a nanoscale feature width of ~90 nm, which could, in principle, serve as templates for subsequent electrochemical deposition of metals such as gold for circular-polarization and other capabilities^8^.

## Nanoprecise 3D metastructures for visible-wavelength all-optical machine learning

**Fig. 5:**
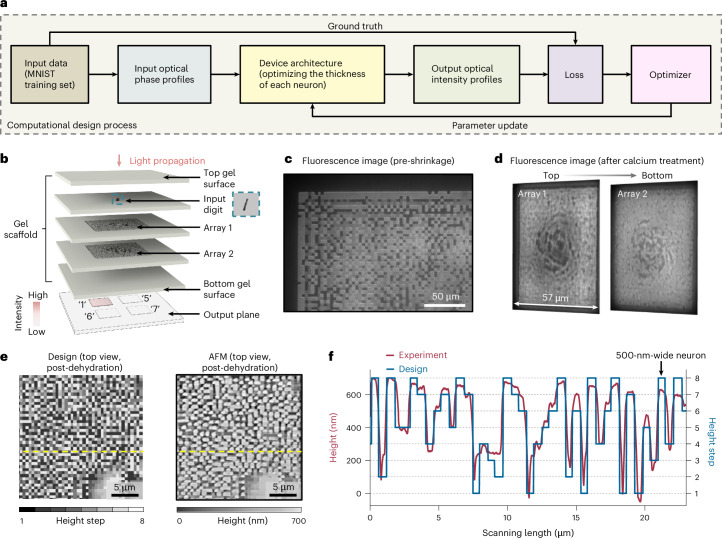
All-optical machine learning devices with nanoscale neuron sizes operating at visible wavelengths. **a**, Computational design process for the device. The device was trained by optimizing the thickness of each neuron to map input digits (from the MNIST training set, and encoded into optical phase profiles) to corresponding output regions on a designated output plane. The intensity of the latter determined the loss function during the design optimization process. **b**, Schematic illustration of such a device for classification. The input digit (‘1’, ‘5’, ‘6’ or ‘7’ from the MNIST test set; vacancies of physical shape based on the physical shape of the digits) and the device were designed to be fabricated within one hydrogel scaffold. The output plane, where the optical intensity profiles were captured by a camera, was designed to be outside the scaffold, with four designated regions, each corresponding to one digit. Blue inset: magnified top-view schematic of the input digit ‘1’ as an example. **c**, Representative sum-intensity projection of *z*-stacked fluorescence images (*n* = 2 areas from 2 devices from 2 gels) of a partial section of one device array in swollen hydrogel (MSF hydrogels were used throughout this figure). **d**, Representative sum-intensity projections of *z*-stacked fluorescence images (*n* = 48 devices from 3 gels) of each array after calcium treatment with an approximately fourfold shrinkage factor. **e**, Design (left) and representative AFM image (right, *n* = 2 areas from 2 arrays from 2 gels; AFM scanning area, ~23 × 23 µm) of a partial section of one array post-dehydration. The structure was initially fabricated within the hydrogel, and the hydrogel above the structure was cleaved to expose the surface to AFM. **f**, Height profiles along the yellow dashed scanning lines in **e**. Experimental results (red line) show strong correspondence to the design (blue line), revealing neurons with lateral dimensions of approximately 500 nm, 8 discrete heights from 0 nm to ~700 nm, and sharp edges. Source data

To explore the potential of ImpCarv for optical computing, we developed a nanoprecise 3D metastructure for all-optical machine learning at visible wavelengths. All-optical machine learning devices leverage 3D refractive index distributions within structures to perform specific, pre-trained, machine learning tasks using light diffraction across multiple arrays (here, patterned in planes distributed along the optical axis of the system, all within a single block of hydrogel), with each array comprising numerous neurons (here, a square pixel of defined vacancy thickness, and thus phase shift, with respect to the bulk hydrogel)^30–34,36^. We designed such a device for a classical all-optical machine learning digit classification task, but aiming to realize this with visible light, and nanoscale neuron sizes. We designed the device architecture on a computer (Fig. 5a), optimizing the thickness of each neuron, to control the phase shift in each neuron according to equation (1), using computational algorithms from previous studies^30,52^. Once the design was finalized, the devices were fabricated using ImpCarv. During the design process, the device was trained to map input digits (from the MNIST (Modified National Institute of Standards and Technology) training set^53^, and encoded into optical phase profiles) to corresponding output regions on a designated output plane. The intensity of the latter determined the loss function during the design optimization process^30^ (see Supplementary Note 1 for further details). The device was designed, in the end (Fig. 5b), to consist of two vacancy arrays within the dehydrated gel scaffold (overall lateral dimension of the device, 60 × 60 µm; inter-array axial distance, ~76 µm; gel filling the space between adjacent arrays), each array containing 120 × 120 neurons (lateral neuron dimensions, 500 × 500 nm; axial neuron dimensions, from 0 nm to ~700 nm with 8 steps) for a target wavelength of 532 nm (additional details on neuron dimension selection are provided in Supplementary Note 2 and Supplementary Figs. 12–14). In addition to the machine learning arrays, we also fabricated the input digit (that is, ‘1’, ‘5’, ‘6’ or ‘7’ from the MNIST test set^53^; vacancies of physical shape based on the physical shape of the digits) within the hydrogel scaffold (schematized in Fig. 5b) for simplicity. Fabricating input digits separately from the machine learning arrays is possible, with precise alignment^30^, but given that our goal was simply to demonstrate a known principle—all-optical machine learning—with visible light and nanoscale neuron sizes, we elected to keep the experiment simple. The output plane, where the optical intensity profiles were captured by a camera, was designed to be outside the scaffold, with four designated regions, each corresponding to one digit. We used the MSF hydrogel to construct the device because it exhibited specifications sufficient to create neurons with desired nanoscale sizes and sharp edges (see AFM results below). We evaluated the device structure in swollen hydrogel (Fig. 5c; sum-intensity projection of *z*-stacked fluorescence images of a partial section of one array), and then checked the structure after calcium treatment with an approximately fourfold shrinkage factor (Fig. 5d; sum-intensity projections of *z*-stacked fluorescence images of each array of the device). To characterize the structural accuracy of the device, we fabricated a single array of the device within the hydrogel and then exposed it to AFM by cleaving the hydrogel above the array (Fig. 5e, with further visualization in Supplementary Fig. 15). We examined a partial section of the array post-dehydration (a scanning area of ~23 × 23 µm in AFM) and compared it with the design. The experimental results (red, Fig. 5f) closely matched the design (blue) along the yellow dashed line in Fig. 5e, revealing neurons with a lateral dimension of approximately 500 nm, 8 discrete heights from 0 nm to ~700 nm, and sharp edges.

**Fig. 6:**
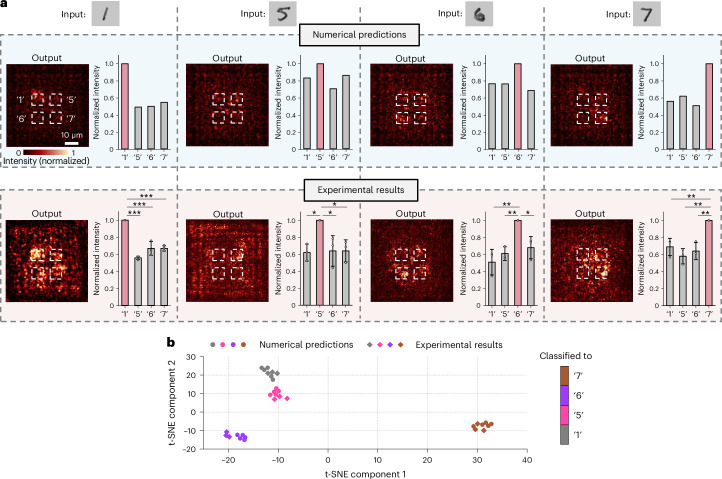
Classification at the visible wavelength. **a**, Classification of several input digits (‘1’, ‘5’, ‘6’ and ‘7’) at a visible wavelength (532 nm). The measured average intensity values for each output region, normalized to the maximum (bottom row in **a**), closely matched the numerical predictions based on the design (top row in **a**). Each column represents the results for a specific input digit, with the top left and bottom left sections of each column showing the numerically predicted and representative experimental output intensity profiles (for each input digit, *n* = 3 devices from 2 gels), respectively (four dashed frames indicate the four target output regions). The top right and bottom right sections show predicted data and experimental statistical data. In both numerical predictions and experimental results, the red bar indicates the highest intensity compared with the grey bars. The average intensities, normalized to the maximum, across four output regions were analysed using one-way ANOVA followed by post hoc Tukey’s HSD tests. For input digit ‘1’, one-way ANOVA, *F*(3, 8) = 47.84, *P* = 0.000018. Post hoc Tukey’s HSD test versus the intensity in output region ‘1’: output region ‘5’, *P* = 0.000016; output region ‘6’, *P* = 0.00013; output region ‘7’, *P* = 0.00015 (first column in **a**). Similarly, for input digit ‘5’, one-way ANOVA, *F*(3, 8) = 6.77, *P* = 0.0138. Post hoc Tukey’s HSD test versus the intensity in output region ‘5’: output region ‘1’, *P* = 0.0210; output region ‘6’, *P* = 0.0289; output region ‘7’, *P* = 0.0289 (second column in **a**). For input digit ‘6’, one-way ANOVA, *F*(3, 8) = 12.16, *P* = 0.00238. Post hoc Tukey’s HSD test versus the intensity in output region ‘6’: output region ‘1’, *P* = 0.00209; output region ‘5’, *P* = 0.00773; output region ‘7’, *P* = 0.02269 (third column in **a**). For input digit ‘7’, one-way ANOVA, *F*(3, 8) = 14.74, *P* = 0.00127. Post hoc Tukey’s HSD test versus the intensity in output region ‘7’: output region ‘1’, *P* = 0.00857; output region ‘5’, *P* = 0.00132; output region ‘6’, *P* = 0.00352. *P < 0.05, **P < 0.01, ***P < 0.001 (fourth column in **a**). **b**, t-SNE visualization of the intensity outputs. The axes represent the first two principal components in t-SNE. Circular points represent the numerically predicted intensity outputs from 5 instances of each digit, picked randomly from the MNIST test set, which contains 1,000 instances of each digit, and rhombic points represent the experimental intensity outputs using 1 randomly chosen instance of a digit, chosen from the 5 (*n* = 3 devices from 2 gels). Each numerically predicted or experimental intensity output was encoded as a four-dimensional vector (*v*_1_, *v*_5_, *v*_6_ and *v*_7_), where *v*_*x*_ denotes the average intensity in the output region ‘*x*’, normalized to the maximum. Colours correspond to the device classification of these input digits (grey, pink, purple and brown for input digits ‘1’, ‘5’, ‘6’ and ‘7’, respectively). Source data

We evaluated the classification performance of the device, using input digits ‘1’, ‘5’, ‘6’ and ‘7’ from the MNIST test set^53^ (Fig. 6a). The experimental results (bottom row in Fig. 6a) showed strong correspondence with the designed outputs (top row in Fig. 6a). Specifically, for each input digit, the device successfully directed the majority of light to the target output region, with clear differentiation from other regions. The average intensities, normalized to the maximum, across the four output regions were analysed using one-way analysis of variance (ANOVA) followed by post hoc Tukey’s honest significant difference (HSD) test (see statistical details in the legend of Fig. 6a). The analysis confirmed that the intensity in the target output region was significantly higher than in the other three output regions. To assess whether these experimental intensity outputs formed distinct classified groups, as designed, we projected both the numerically predicted intensity outputs (circular points, Fig. 6b, highlight predictions from 5 instances of each digit, picked randomly from the MNIST test set, which contains 1,000 instances of each digit^53^) and experimental intensity outputs (rhombic points, Fig. 6b, using 1 randomly chosen instance of a digit, chosen from the 5, to result in *n* = 3 devices from 2 gels for each digit) into a two-dimensional (2D) space using t-distributed stochastic neighbour embedding (t-SNE)^54^ (Fig. 6b). The numerical predictions formed four distinct areas corresponding to the four input digits (grey, pink, purple and brown for input digits ‘1’, ‘5’, ‘6’ and ‘7’, respectively), with experimental results and numerical predictions clustering together. Together, these results demonstrated that the device successfully classified input digits at visible wavelengths, and also proved the utility of ImpCarv in fabricating such nanoprecise 3D metastructures. Moreover, ImpCarv allows structures with an increased number of arrays (one seven-array structure is shown in Supplementary Fig. 16) and larger overall dimensions (one half-millimetre structure is shown in Supplementary Fig. 17). Overall, ImpCarv holds promise for a wide range of nanophotonic applications^30–34^ and beyond^55^.

## Conclusion

We here enable the fabrication of 3D metastructures, with resolution in the tens of nanometres, capable of supporting visible light applications. With such 3D nanoprecise refractive index programmability and resulting precise phase control, we demonstrated an all-optical machine learning device with nanoscale neuron sizes that can operate at visible wavelengths. ImpCarv could, in principle, be integrated with photopatterning of materials as in implosion fabrication^4,21^ at defined points throughout nanoprecise 3D metastructures, for applications such as nonlinear control in optical computing by depositing saturable absorbers^56^, which could potentially enable broad nonlinear machine learning tasks. Furthermore, the refractive index contrast in ImpCarv could, potentially, be enhanced by increasing the refractive index of the hydrogel through the incorporation of additional, specific, materials^57^. While established semiconductor-based optical processing units are powerful^58^, they are constrained on 2D or 2.5D silicon architectures. ImpCarv, by contrast, enables the fabrication of monolithic 3D metastructures operating in free space. By enabling the programming of a complex, volumetric refractive index landscape, it opens pathways for high-density 3D optical computation, nanophotonics and beyond.

We envision ImpCarv as a scalable and cost-effective platform for fabricating nanoprecise 3D metastructures (see details in Supplementary Note 3). First, the materials used in our approach are commercially available and cost-effective. Unlike other photocleavable methods, such as those based on nitrobenzyl ether moieties^13,14^ or ruthenium polypyridyl complexes^15,16^, ImpCarv can support optics and is applicable to diverse hydrogel compositions. Although 2D planar nanostructures with multiple height steps could be fabricated using lithographic nanofabrication methods that require multiple cycles of nanoprecise alignment^59^, ImpCarv offers a simple alternative with a single-shot photopatterning process that eliminates the need for alignment steps. Such a single-shot process enables the creation of not only 2D but also 3D structures, which are challenging in lithographic nanofabrication methods. Lastly, while our current fabrication speed is limited by the use of a conventional two-photon microscope that is not optimized for photopatterning, there are several potential approaches to enhance the photopatterning speed. One approach is to replace point-scanning two-photon microscopes by line-scanning ones^4,5^. Deploying light-sheet microscopes instead of two-photon microscopes offers another promising approach^60^. These approaches could hugely improve the scalability and lower the cost of ImpCarv.

## Methods

### Overview

Our standard workflow consists of the following steps: (1) hydrogel synthesis, (2) sample immersion in a photosensitizer solution, (3) photopatterning using a two-photon microscope, (4) shrinkage through treatment with monovalent and divalent cation solutions, (5) solvent exchange from the divalent cation solution to ethanol and (6) dehydration via supercritical drying. The experimental procedures associated with each figure are detailed in Supplementary Table 3. All immersions in solutions, except during the photopatterning and shrinkage processes, were conducted on an orbital shaker set to 80 revolutions per minute; immersions during the shrinkage process were performed on an orbital shaker set to 200 revolutions per minute. Unless otherwise specified, all experiments were carried out at room temperature.

### Materials

Unless otherwise specified, all chemicals were purchased from Sigma-Aldrich and used without further purification. The starting materials for the hydrogels included sodium acrylate (Gelest), acrylamide, *N*,*N*′-methylenebisacrylamide (MBAA), ammonium persulfate (APS), tetramethylethylenediamine (TEMED), agarose, methacrylated alginate (Advanced Biomatrix), methacrylated gelatin (Advanced Biomatrix) and 2-hydroxy-1-[4-(2-hydroxyethoxy)phenyl]-2-methyl-1-propanone (Irgacure D-2959). The photosensitizers used were rhodamine B, methylene blue and rhodamine 123. The chemicals used for photopatterning and dehydration included isopropylamine, H_2_O_2_, NaCl, MgCl_2_, CaCl_2_ and ethanol.

### Hydrogel synthesis process

Detailed synthesis procedure steps for the hydrogels were previously described^21,61^. In brief, the synthesis of HSF hydrogels began with the preparation of solution A by dissolving sodium acrylate (26.0 w/v%), acrylamide (7.5 w/v%) and phosphate-buffered saline (PBS; 10×; 16.7 vol%) in UltraPure water (UltraPure DNase/RNase-free distilled water; Thermo Fisher Scientific). Solutions B–D were prepared by dissolving *N*,*N*′-methylenebis(acrylamide) (MBAA, 2.0 w/v%), ammonium persulfate (APS, 10.0 w/v%) and tetramethylethylenediamine (TEMED, 10.0 vol%) in UltraPure water, respectively. Solution E was created by mixing solution A (600 µl), solution B (13 µl) and UltraPure water (347 µl), followed by bubbling with N_2_ for 3 min. Subsequently, 192 µl of solution E was mixed with 4 µl of solution D and 4 µl of solution C, in sequence. The resulting mixture was cast into a hydrophobic mould (see below for details about the mould preparation; dimensions, ~20 mm in length, ~10 mm in width and ~0.17 mm in depth). The moulds consisted of a glass slide as the base and a no. 1.5 glass coverslip as the top, with glass coverslips used as the spacer on two sides, one for each side. The hydrophobic surfaces of the mould were prepared by pre-immersing the glass slide and glass coverslips in a solution of trichloro(octadecyl)silane (0.2 vol% in hexane) for 90 s, followed by rinsing with ethanol and double-distilled water (ddH_2_O) 3 times each, each for 30 s. The hydrophobic mould, with the hydrogel mixture inside, was placed in a 37 °C oven for at least 3 h for gelation. The hydrogels were then washed 3 times in ddH_2_O, each for 30 min. Throughout the ImpCarv process, all solutions used with unlisted volumes were applied in large excess relative to the hydrogel volume.

The synthesis of MSF hydrogels followed a similar procedure, with modifications to solutions A, E and the mould dimensions. The modified solution A contained sodium acrylate (39.0 w/v%), acrylamide (11.3 w/v%) and PBS (10×; 16.7 vol%) in UltraPure water. Solution E was prepared by mixing the modified solution A (600 µl), solution B (120 µl) and UltraPure water (240 µl), followed by bubbling with N_2_ for 3 min. Except for the mould dimensions, the remaining procedures were identical to those used for the HSF hydrogels. The mould consisted of a glass slide as the base and a no. 1.5 glass coverslip as the top, with two additional glass coverslips as spacers on either side (dimensions, ~20 mm in length, ~10 mm in width and ~0.34 mm in depth).

For agarose hydrogel synthesis, agarose (5.0 w/v%) was dissolved in UltraPure water, microwaved for 2 min and then cast into a hydrophobic glass mould to cool slowly at room temperature. For alginate or gelatin hydrogel synthesis, methacrylated alginate (5.0 wt%) or methacrylated gelatin (2.0 w/v%) was dissolved in UltraPure water, followed by the addition of Irgacure D-2959 (0.25 w/v%). The solution was then cast into a hydrophobic glass mould and exposed to ultraviolet light (wavelength, 365 nm) for 60 min for gelation. Since these hydrogels do not exhibit considerable expansion or shrinkage, the moulds were constructed with a glass slide as the base and a no. 1.5 glass coverslip as the top, using four additional glass coverslips as spacers on either side (dimensions, ~20 mm in length, ~10 mm in width and ~0.68 mm in depth). The hydrogels were then washed 3 times in ddH_2_O, each for 30 min.

### Immersion process in a photosensitizer solution

After washing with ddH_2_O, the hydrogels were cut into ~1.5 × 1.5 cm squares, transferred into a glass-bottom dish (D60-30-1-N; Cellvis) and immersed in ~5 ml of photosensitizer solution (details below) for 30 min. Following immersion, a 40-mm-diameter glass coverslip (Bioptechs) was placed over the well of the glass-bottom dish containing the hydrogel sample, and excess photosensitizer solution was removed.

For the samples shown in Figs. 2b,c,e,g and 3b–j and Supplementary Figs. 3–5, 11 and 16, the photosensitizer solution consisted of 150 µM rhodamine B, 10 mM H_2_O_2_ and isopropylamine, maintaining the pH of the solution at ~8.0–9.5. Before use, the photosensitizer solution was bubbled with O_2_ for 5 min to enhance photocleavage efficiency (see the results about photocleavage efficiency enhancement in Supplementary Fig. 1). For the samples shown in Figs. 4b–d, 5c–f and 6a, and Supplementary Figs. 1b, 10, 15 and 17, the photosensitizer solution consisted of 250 µM rhodamine B, 10 mM H_2_O_2_ and isopropylamine, maintaining the pH of the solution at ~8.0–9.5. Before use, the solution was bubbled with O_2_ for 5 min. For the samples shown in Supplementary Fig. 1c, the photosensitizer solution consisted of 250 µM rhodamine B and isopropylamine, maintaining the pH of the solution at ~8.0–9.5. Before use, the solution was bubbled with O_2_ for 5 min. For the samples shown in Supplementary Fig. 1d, the photosensitizer solution consisted of 250 µM rhodamine B and isopropylamine, maintaining the pH of the solution at ~8.0–9.5. Before use, the solution was bubbled with N_2_ for 5 min. For the samples shown in Supplementary Fig. 6g–i, the photosensitizer solution consisted of 1 mM rhodamine B, 10 mM H_2_O_2_ and isopropylamine, maintaining the pH of the solution at ~8.0–9.5. Before use, the solution was bubbled with O_2_ for 5 min. For the samples shown in Supplementary Fig. 7d, the photosensitizer solution consisted of 250 µM methylene blue, 10 mM H_2_O_2_ and isopropylamine, maintaining the pH of the solution at ~8.0–9.5. Before use, the solution was bubbled with O_2_ for 5 min. For the samples shown in Supplementary Fig. 7e, the photosensitizer solution consisted of 250 µM rhodamine 123, 10 mM H_2_O_2_ and isopropylamine, maintaining the pH of the solution at ~8.0–9.5. Before use, the solution was bubbled with O_2_ for 5 min. For the samples shown in Supplementary Fig. 8, the photosensitizer solution consisted of 150 µM rhodamine B.

### Photopatterning process

The photopatterning process was conducted using either a custom-built two-photon laser system (Mai Tai Ti laser; wavelength, 780 nm and 1,050 nm; pulse width, 100 fs; frequency, 80 MHz) or an inverted Zeiss LSM 710 confocal microscope equipped with a Chameleon Ultra II femtosecond pulsed infrared laser (wavelength, 780 nm; pulse width, 140 fs; frequency, 80 MHz). The custom two-photon laser system used a water-immersion objective with an NA of 1.00 (CFI75 Apochromat LWD 20XC; working distance, 2.80 mm) and a beam expander to fill the back aperture of the objective, while the Zeiss LSM 710 used a water-immersion objective with an NA of 1.24 (CFI Apochromat Lambda S 40XC; working distance, 0.18 mm). Photopatterning parameters in each experiment, including average laser power, dwell time and *z*-step size, were adjusted according to each sample condition (see details of these photopatterning parameters in Supplementary Table 3). Rhodamine photosensitizers (rhodamine B and rhodamine 123) required a 780 nm wavelength for two-photon laser excitation, while methylene blue required a 1,050 nm wavelength owing to its distinct absorption properties compared with rhodamine photosensitizers^62^.

For photopatterning in Fig. 2b,c and Supplementary Fig. 4, the custom two-photon laser system was operated at laser powers ranging from 15 mW to 33 mW, a dwell time of 6 µs and a *z*-step size of 1 µm. These structures were initially generated inside the hydrogel at different laser powers; the hydrogel above the photopatterned regions was subsequently removed using a higher laser power (40 mW; dwell time, 6 µs; *z*-step size, 1 µm) for imaging purposes. For photopatterning in Figs. 2e,g and 3b–j and Supplementary Figs. 3 and 5, the custom two-photon laser system was operated at a laser power of 30 mW, a dwell time of 6 µs and a *z*-step size of 250 nm. For Fig. 3b–j, these structures were initially generated inside the hydrogel; the hydrogel above the photopatterned regions was subsequently removed using a higher laser power (40 mW; dwell time, 6 µs; *z*-step size, 1 µm) for imaging purposes. For photopatterning in Fig. 4b–d, the custom two-photon laser system was operated at laser powers of 90 mW and 100 mW, a dwell time of 4 µs and a *z*-step size of 0.75 µm or 1.0 µm. These structures were initially generated inside the hydrogel; the hydrogel above the photopatterned regions was subsequently removed (100 mW; dwell time, 4 µs; *z*-step size, 0.75 µm or 1.0 µm) for imaging purposes. For photopatterning in Fig. 5e,f and Supplementary Fig. 15, the custom two-photon laser system was operated at a laser power of 100 mW, a dwell time of 3 µs and a *z*-step size of 0.5 µm. These structures were initially generated inside the hydrogel; the hydrogel above the photopatterned regions was subsequently removed (100 mW; dwell time, 3 µs; *z*-step size, 1 µm) for imaging purposes. For photopatterning in Figs. 5c,d and 6a, the custom two-photon laser system was operated at a laser power of 80 mW, a dwell time of 3 µs and a *z*-step size of 0.5 µm. For photopatterning in Supplementary Figs. 1b–d and 7d,e, the Zeiss LSM 710 was operated at laser powers ranging from 2 mW to 109 mW, a dwell time of 0.79 µs and a *z*-step size of 2.0 µm. For photopatterning in Supplementary Fig. 6g–i, the custom two-photon laser system was operated at a laser power of 200 mW, a dwell time of 6 µs and a *z*-step size of 1.0 µm. These structures were initially generated inside the hydrogel. The hydrogel above the photopatterned regions was subsequently removed (200 mW; dwell time, 6 µs; *z*-step size, 1 µm), with an initial goal for SEM or AFM imaging; however, owing to the anisotropic hydrogel deformation during the ethanol solvent exchange, it was not feasible to dehydrate these gels while preserving the structures. As a result, fluorescence imaging in an aqueous solution (for example, PBS (1×)) was used instead. For photopatterning in Supplementary Fig. 8, the custom two-photon laser system was operated at laser powers ranging from 60 mW to 150 mW, dwell times of 1 µs and 2 µs, and a *z*-step size of 2.0 µm. For photopatterning in Supplementary Fig. 10, the custom two-photon laser system was operated at laser powers of 60 mW, 70 mW, 80 mW, 90 mW and 100 mW, a dwell time of 4 µs and a *z*-step size of 0.2 µm. For photopatterning in Supplementary Fig. 11, the custom two-photon laser system was operated at a laser power of 30 mW, a dwell time of 6 µs and a *z*-step size of 0.5 µm. For photopatterning in Supplementary Fig. 16, the custom two-photon laser system was operated at a laser power of 30 mW, a dwell time of 6 µs and a *z*-step size of 1.7 µm. For photopatterning in Supplementary Fig. 17, the custom two-photon laser system was operated at a laser power of 100 mW, a dwell time of 3 µs and a *z*-step size of 1.0 µm.

### Shrinking process involving sodium–magnesium treatment and calcium treatment

Before the shrinking process, the hydrogels were washed in an aqueous isopropylamine solution (pH ~8.0–9.5) 4 times, each for 30 min. The initial step of the shrinking process involved treatment with NaCl solution at a concentration of 0.02 M for 15 min, and then sequential treatment with MgCl_2_ solutions at concentrations of 0.1 M, 0.3 M and 0.5 M, each for 30 min (we call this sodium–magnesium treatment). All solutions were filtered using polyvinylidene fluoride (PVDF) syringe filters with a pore size of 200 nm before use. Following the treatment with 0.5 M MgCl_2_, the hydrogels were further shrunk using sequential treatments with CaCl_2_ solutions at concentrations of 0.1 M, 0.3 M and 0.5 M, each for 30 min (we call this calcium treatment), which were also filtered with PVDF syringe filters (pore size, 200 nm).

### Ethanol solvent exchange and supercritical drying

After calcium treatment, the hydrogels were immersed in 0.5 M CaCl_2_ solution with a volume of ~5 ml, in large excess relative to the hydrogels. Subsequently, two syringe pumps (Fusion 200-X Syringe Pump, Chemyx) were used to infuse a high-concentration CaCl_2_ solution (5.0 M; filtered with PVDF syringe filters; pore size, 200 nm) at a rate of 0.25 ml h^−1^, while keeping the total volume constant (removal rate, 0.25 ml h^−1^) for a period of 48 h (estimated CaCl2 concentration after infusion and removal, ~4.6 M). Next, the hydrogels were incubated in 4.8 M and 5.0 M CaCl_2_ for 30 min each (both solutions were filtered with PVDF syringe filters; pore size, 200 nm). Then, we gradually replaced the 5.0 M CaCl_2_ solution with ethanol. Following the treatment with 5.0 M CaCl_2_ solution, we manually replaced the solution with ethanol in a stepwise manner, increasing the ethanol concentration from 0 vol% to 100 vol% in increments of 5 vol% within the 5.0 M CaCl_2_–ethanol mixture (0 vol%, 5 vol%, 10 vol%, 15 vol%, 20 vol%, … 80 vol%, 85 vol%, 90 vol%, 95 vol% and 100 vol%, 30 min each). After the solvent exchange, the scaffold was immersed in pure ethanol 3 times, each for 30 min. The hydrogels were then placed into microporous specimen capsules (pore size, 78 µm; Electron Microscopy Sciences) while still immersed in ethanol. Supercritical drying was performed using a critical point dryer (Autosamdri-931, Tousimis) with a default parameter set (fill time, 150 s; purge time, 600 s; post-purge time, 90 s; critical point duration, 120 s).

### Device design

The architecture of the all-optical machine learning device was designed using computational algorithms based on previous studies^30,52^. The device was trained using the MNIST training set (all the images corresponding to 4 digits (‘1’, ‘5’, ‘6’ and ‘7’) were used; 24,000 images in total; 6,000 images per digit). Of these, 22,000 images (5,500 per digit) were used for training, while 2,000 images (500 per digit) were used for validation. Testing was conducted using the MNIST test set (all the images corresponding to the 4 digits were used; 4,000 images in total; 1,000 images per digit). Training was performed with the Adam optimizer, with a learning rate of 0.003. The device achieved the desired mapping functions between the input and the output after 18 epochs. The entire design process was conducted on a desktop system equipped with an NVIDIA DGX-1 architecture (CPU, Dual 20-Core Intel Xeon E5-2698 v4 at 2.2 GHz; GPU, Tesla V100 with 16 GB memory; system memory, 512 GB DDR4 LRDIMM at 2,133 MHz). TensorFlow 1.x (Google) was used as the machine learning framework in the design.

### Imaging

All fluorescence images were acquired using a PerkinElmer spinning disk confocal microscope (CSU-10 Yokogawa) equipped with a Hamamatsu Orca-ER cooled CCD camera. A 40× Nikon N40XLWD-NIR water-immersion objective with an NA of 1.15 and a working distance of 0.59–0.61 mm was used.

All optical images, except for Supplementary Fig. 3a, were obtained using a Nikon ECLIPSE Ts2 with bright-field illumination. We utilized diverse objectives for optical imaging. Supplementary Fig. 3a was taken by a smartphone camera.

All SEM images were captured using a Gemini 360 FE-SEM (ZEISS) equipped with an Energy Selective Backscatter detector and an Everhart–Thornley secondary electron detector. Before imaging, the sample surface was coated with a 10-nm-thick layer of Pd/Pt using an EMS 150T ES Sputter/Carbon Coater.

All AFM images were captured using a Cypher VRS AFM (Oxford Instruments) with an AFM tip (NW-AR5T-NCHR from NanoAndMore or AC160TS-R3 from Oxford Instruments; chosen randomly). The images were taken in tapping mode in the air.

All phase images were captured using a custom-built diffraction phase microscope. The system consisted of a supercontinuum source (Fianium Whitelase SC480) collimated and filtered to provide on-axis plane wave illumination on the sample at 532 nm or 633 nm. The perturbed sample field was collected by a 20×, 1.00 NA water-immersion objective (Olympus, XLUMPLFLN20XW) and passed through a 160 lp mm^−1^ Ronchi ruling to replicate the measured field into 0th order, +1st order and −1st order beams. The 0th order field was filtered with a 5-μm-diameter pinhole to recover the original incident plane wave as a reference while the −1st order field was blocked to enable interference between the plane wave and +1st order field. The recombined field was magnified through a 4× 4F relay lens configuration to generate a sufficiently sampled interferogram at the camera (Optronis, CP90-25P-M-72). The phase results were subsequently recovered by applying a Hilbert transform to the measured interferogram^63^.

For the device function evaluation, we also utilized the custom-built diffraction phase microscope. For this evaluation, the filtered 0th order reference beam was blocked to prevent interferogram formation and measure only the perturbed field’s intensity distribution. In this configuration, the diffraction phase microscope operated as a bright-field microscope with plane wave illumination at normal incidence.

### Analysis

For the measurements of lateral feature sizes shown in Fig. 3d–f, ImageJ was used. The images were rotated to orient the lines vertically, and the mean pixel value was calculated over the vertical dimension for a clean line segment. The FWHM was calculated using Gaussian single peak fitting. Any cracked lines observed during sample preparation were excluded from the analysis as they did not represent the intended trench lines.

For the measurements of axial feature sizes and surface roughness values shown in Fig. 3h–j and Supplementary Figs. 5 and 15, Gwyddion, an open-source software for AFM image processing, was used. For Fig. 3i, the images were rotated to align the stepped structures horizontally, and the mean pixel value was calculated along the vertical dimension to obtain a clean step segment. The average step height (Fig. 3j) and surface root-mean-square roughness (Supplementary Fig. 5) were measured over an ~0.5 × 2.0 µm and ~1.0 × 1.0 µm windows, respectively, using the moment-based function in Gwyddion. For Fig. 3i, the images were rotated to align the stepped structures horizontally, and the mean pixel value was calculated along the vertical dimension to obtain a clean step segment.

For the phase contrast measurements shown in Fig. 4 and Supplementary Fig. 8, we recovered the results utilizing a Hilbert transform (code available in GitHub). The transform consisted of applying a 2D fast Fourier transform to the measured interferogram, applying a Pupil filter to extract the encoded field information, and applying an inverse 2D fast Fourier transform. The log of the field magnitude was used to extract the absorption, and the phase angle was extracted and unwrapped across the whole interferogram. In Fig. 4b,d, the post-dehydrated gels were immersed in index-similar oil (refractive index, 1.48) to reduce the index contrast at the interface and thereby minimize noise during phase imaging^64^. The $\varphi$ values were obtained by averaging a collection of pixels in the central region (diameter, ~5 µm) of each circle in the phase image and then subtracting the background reference. Patterns with surface dust contamination were excluded from analysis, as they did not represent the intended phase results. The grey dashed line represents the linear fitted results, with the slope indicating a refractive index of ~1.5, based on the equation $\Delta \varphi =(2\pi /\lambda )\times \Delta n\times \Delta h$, where $h$ is the AFM-measured depth of these circular patterns and $\lambda$ is the wavelength (532 nm). In Supplementary Fig. 8, the refractive index contrast, $\Delta n$, was directly calculated using the equation $\Delta \varphi =(2\pi \times \Delta n\times h)/\lambda$, where $h$ represents the depth of vacancies (obtained by dividing designed depth by the shrinkage factor; the focal depth of the laser in a multi-photon system was neglected because it was much smaller than the designed depth) and $\lambda$ is the wavelength of light (633 nm).

For the device function evaluation in Fig. 6a, we obtained the intensity distribution at the four output regions (area for each output region, ~9.5 × 9.5 µm) for a visible wavelength of 532 nm, and then utilized a custom MATLAB algorithm to calculate the average intensity in each output region (code available in GitHub). One-way ANOVA was utilized for the analysis, followed by the post hoc Tukey’s HSD test reported in the legend of Fig. 6a. *P* < 0.05 was considered statistically significant. These statistical analyses were performed using Prism software (GraphPad).

For t-SNE visualization in Fig. 6b, each numerically predicted or experimental intensity output was encoded as a four-dimensional vector (*v*_1_, *v*_5_, *v*_6_ and *v*_7_), where *v*_*x*_ denotes the average intensity in the output region ‘*x*’, normalized to the maximum. These four-dimensional vectors were visualized in 2D using t-SNE, a general dimensionality reduction algorithm, to represent the similarity of each categorized data point (code available in GitHub). The t-SNE was implemented with the open-source scikit-learn machine learning library.

For the geometry measurements in Fig. 2b and Supplementary Fig. 4, ImageJ was used. Depth measurements were obtained from the side-view fluorescence image by *z*-staking fluorescence images along the grey dashed line in Supplementary Fig. 4a. From the side-view fluorescence images, we measured the fluorescence intensity along the axial direction in regions 1 and 2 (Supplementary Fig. 4b) and fitted these curves of fluorescence intensity versus scanning depth using a double Gaussian distribution (Supplementary Fig. 4c). The distance between the peaks of the two curves was used to determine the depth of the circular hole. For diameter measurements, we extracted the fluorescence intensity from a single *z*-slice fluorescence image along the white dashed line in the inset of Supplementary Fig. 4e, and the FWHM calculation was manually performed to determine the diameter.

For the shrinkage factor measurement presented in Supplementary Fig. 2, we calculated the shrinkage factors by comparing the design dimensions with the structural dimensions at different stages using fluorescence, optical and phase imaging. The samples were selected based on the availability of high-resolution fluorescence, optical and phase images at various stages and were sourced from multiple experiments.

## Online content

Any methods, additional references, Nature Portfolio reporting summaries, source data, extended data, supplementary information, acknowledgements, peer review information; details of author contributions and competing interests; and statements of data and code availability are available at 10.1038/s41566-026-01896-1.

## Supplementary information

Supplementary Information Supplementary Notes 1–3, Figs. 1–17 and Tables 1–3.

## Source data

Source Data Fig. 2 Depth and diameter data in Fig. 2b.

Source Data Fig. 3 Inverse greyscale profile data in Fig. 3e, FWHM statistical data in 3f, AFM height data in 3i and step height statistical data in Fig. 3j.

Source Data Fig. 4 AFM depth data in Fig. 4c and phase versus depth data in Fig. 4d.

Source Data Fig. 5 Scanning height data in Fig. 5f.

Source Data Fig. 6 Intensity output data in Fig. 6a and t-SNE data in Fig. 6b.

## Data Availability

Source data are provided with this paper. These data and raw images associated with this study are available via figshare at https://figshare.com/s/5cc9870df36c3852fd39 (ref. ^65^).

## Code availability

The reconstruction code for phase measurement is available at https://github.com/matlockCISL/DPM-basic. The functional analysis code for all-machine learning devices and the t-SNE code are available at https://github.com/TakahiroNambara/NanoVac.
