# Supplementary material for: Isotropic shrinkage of patterned vacancies enables three-dimensional nanoprecise metastructures for visible light applications
Source: Nat Photonics. 2026 May 12;20(6):653–63. doi: 10.1038/s41566-026-01896-1 (PMC13241318; doi:10.1038/s41566-026-01896-1)
Supplement: Supplementary file 1 — Supplementary Notes 1–3, Figs. 1–17 and Tables 1–3. [file 41566_2026_1896_MOESM1_ESM.pdf]

nature photonics

Supplementary information

https://doi.org/10.1038/s41566-026-01896-1

Isotropic shrinkage of patterned vacancies enables three-dimensional nanoprecise metastructures for visible light applications

In the format provided by the authors and unedited

Table of Contents

Supplementary Note 1: Equations used in computational design process 2

Supplementary Note 2: Neuron dimension selection by considering the light propagation within array using the finite-difference time-domain (FDTD) method 3

Supplementary Note 3: ImpCarv as a scalable and cost-effective platform for fabricating nanoprecise 3D metastructures 7

Supplementary Fig. 1: Photopatterning under different laser powers and different conditions of photosensitizer solution 9

Supplementary Fig. 2: Dehydration process and shrinkage factors for HSF and MSF hydrogels at different stages 10

Supplementary Fig. 3: Appearance of samples post-dehydration 11

Supplementary Fig. 4: Geometry measurements of circular holes based on fluorescence intensity analysis 12

Supplementary Fig. 5: Root-mean-square roughness of the surface in photopatterned regions with a laser power above threshold (*i.e.*, 30 mW) measured by AFM 13

Supplementary Fig. 6: Versatility for diverse hydrogel scaffolds 14

Supplementary Fig. 7: Versatility for diverse photosensitizers 15

Supplementary Fig. 8: Refractive index contrast (Δn) after sodium-magnesium treatment 16

Supplementary Fig. 9: Index-matching method to examine the refractive index of the post-dehydrated gels 17

Supplementary Fig. 10: 3D photonic crystals with nanoprecise structures indicating a photonic bandgap in the visible spectral range 19

Supplementary Fig. 11: 3D vacant spirals with nanoprecise structures 21

Supplementary Fig. 12: Phase distribution and shifts in half-λ-sized neurons 22

Supplementary Fig. 13: Impact of neuron size on refractive indices and diffraction angles 23

Supplementary Fig. 14: Phase distributions and error analysis 24

Supplementary Fig. 15: Additional AFM measurement on the device structures 25

Supplementary Fig. 16: Seven-array 3D metastructures 26

Supplementary Fig. 17: Large-area devices with half-millimeter-level overall dimensions achieved by stitching multiple photopatterned regions 27

Supplementary Table 1: Formulation of the HSF hydrogel 28

Supplementary Table 2: Formulation of the MSF hydrogel 28

Supplementary Table 3: Summary of experimental procedures used to generate each figure 29

References 32

Supplementary Note 1: Equations used in computational design process

The computational design process incorporates phase modulation calculations within array and free-space light propagation calculations between arrays[1,2].

The equation for phase modulation calculations within array is $u^{out}(x, y) = u^{in}(x, y)\exp(j\Delta\varphi(x, y))$, where $u^{out}(x, y)$ and $u^{in}(x, y)$ are the output and input complex amplitudes of light at position (x, y). $\Delta\varphi(x, y)$ refers to the phase difference at position (x, y), obtained by the equation $\Delta\varphi = (2\pi \times \Delta n \times h(x, y))/\lambda$, the Equation [1] in the main text, where $h(x, y)$ is the depth at position (x, y) in the diffractive array, Δn is the refractive index contrast between vacancies and materials, and λ is the wavelength of light.

The free-space light propagation calculations between arrays were performed using the angular spectrum equation[3], $u(x, y; z = z_0 + d) = \mathcal{F}^{-1}\{\mathcal{F}\{u(x, y; z = z_0)\} \times H(f_x, f_y; d)\}$, where $\mathcal{F}$ and $\mathcal{F}^{-1}$ denote the Fourier transform and its inverse, respectively. $H(f_x, f_y; d)$ is the transfer function for free-space propagation over an inter-array axial distance d, and f_x and f_y represent the Fourier frequencies in the x and y directions, respectively. $H(f_x, f_y; d) = \exp(j2\pi d\sqrt{\lambda^{-2} - f_x^2 - f_y^2})$ where $f_x^2 + f_y^2 \leq \lambda^{-2}$ and $H(f_x, f_y; d) = 0$ otherwise.

Supplementary Note 2: Neuron dimension selection by considering the light propagation within array using the finite-difference time-domain (FDTD) method

The all-optical machine learning devices were designed using half-λ-sized neurons, where λ refers to the wavelength of light (schematized in Supplementary Fig. 12a)[1,4]. This design choice stemmed from the fact that a half-λ-sized neuron represented the finest phase distribution required to control the diffraction angle across the entire range from 0 to 90°. If the neuron size was less than half-λ, the array only generated evanescent light, which did not propagate in the forward direction. The phase modulation calculations using the equation [1] in the main text were applied in the design using half-λ-sized neurons[1,4]. The phase modulation calculations assumed a short propagation distance due to a sufficiently large refractive index contrast (Δn) between the neurons and the surrounding medium, which allowed intra-array propagation effects to be neglected. By neglecting intra-array propagation effects, the design process was considerably simplified and accelerated, reducing the computational complexities and cost (for example, one single design of our whole device in FDTD simulations takes ~ 10,000 years). However, the phase modulation calculations became problematic in realistic Δn regimes, such as Δn values between 0.1 and 1.0, where the array was not sufficiently thin to neglect intra-array propagation effects. Therefore, considerable errors in phase and intensity distribution may arise in such realistic Δn regimes (*e.g.*, when the neurons were composed of polymers with refractive indices ranging from 1.3 to 1.5 and the surrounding medium was air with a refractive index of 1.0).

Here, we performed FDTD simulations using Lumerical FDTD software (Ansys, USA) to optimize the structural design with a goal of minimizing the difference between phase modulation calculations and FDTD simulations, while maintaining sufficient diffraction angle to form optical neural networks between arrays. While FDTD simulations offered higher accuracy than phase modulation calculations by accounting for intra-array propagation effects that the phase modulation calculations neglected, its

computational cost limited it to small neuron clusters within one array. Therefore, we checked the differences between phase modulation calculations and FDTD simulations across various structural designs, to obtain the optimized design with minimized differences. Given that common acrylic polymers have refractive indices ranging from 1.3 to 1.5, corresponding to a Δn of 0.3 to 0.5 relative to air, we selected a representative Δn of 0.4 for the simulations. Such a Δn is different from the value obtained by ImpCarv (Δn = ~0.3); we will talk about the effect of the difference at the end of this section.

We began by simulating light propagation through a single isolated half-λ-sized neuron, shown in Supplementary Fig. 12b. When the height of the neuron was λ, the phase shift obtained by phase modulation calculations was 0.8π; however, the phase shift calculated using FDTD simulations was only 0.4π. This noticeable difference indicated that the phase shift obtained from FDTD simulations, a more precise method, was much smaller than that obtained by phase modulation calculations. Furthermore, we plotted the phase shifts calculated by FDTD simulation against various neuron heights, as shown in Supplementary Fig. 12c. For half-λ-sized neurons with multiple different heights, the phase shifts were obviously smaller than those obtained by phase modulation calculations. Supplementary Figure 9d showed the FDTD simulation results for a half-λ-sized neuron with a height of 2.5λ. Although phase modulation calculations obtained a phase shift of 2π, the FDTD simulation showed only a phase shift of 0.64π. Additionally, a substantial portion of the light intensity was confined within the neuron, which had a higher refractive index than the surrounding medium (*i.e.*, air), as shown in the intensity distribution in Supplementary Fig. 12d. These findings suggested that an isolated neuron could partially behave like a waveguide, confining the incident light within its structure.

Here, we introduced the concept of the effective refractive index[5], commonly used in waveguide studies, to consider the confinement effect. The effective refractive index decreased due to the electric field of light distribution spreading to the surrounding medium, in addition to that being confined within neurons (schematized in Supplementary Fig. 13a). As we plotted the effective refractive indices of neurons with different neuron sizes (Supplementary Fig. 13b), the effective refractive index for a neuron with a size

around half-λ was lower than in the refractive index of the material for neurons (*i.e.*, 1.4). Additionally, the effective refractive index change was steep near the half-λ size, indicating high sensitivity to variations in neuron sizes during fabrication. This was critical for fabrication tolerance, as slight variations in a half-λ-sized neuron could cause abrupt changes in the effective refractive index. Furthermore, we plotted the maximum diffraction angles against neuron sizes (schematized diffraction angle range in Supplementary Fig. 13c and data in Supplementary Fig. 13b). The results indicated that a half-λ-sized neuron could achieve a full 90° diffraction. However, as neuron size increased, the controllable diffraction angle decreased substantially, highlighting a clear trade-off between the effective refractive index and diffraction angle range.

Moving forward, we considered the influence of the effective refractive index within neuron arrays. In such structures, numerous neurons were distributed and organized into clusters of different sizes (schematized in Supplementary Fig. 13d). Some neuron clusters were large, while others were small, resulting in unexpected effective refractive index distributions due to their cluster sizes. Thus, phase shift primarily depended on neuron cluster sizes rather than neuron height, leading to substantial deviations from the intended phase distribution. Therefore, the device performance was primarily determined by the neuron cluster size distribution, raising issues in device design. To investigate such issues, we performed FDTD simulations on a 30×30 neuron arrays under different conditions. Supplementary Figure 11a clearly shows that the array with half-λ-sized neurons (left side) exhibits fewer phase changes than phase modulation calculations (right side). The squared error at each neuron in the array with half-λ-sized neurons, by squaring the phase difference between the phase modulation calculations and the FDTD simulations at each neuron, depicted on the left side of Supplementary Fig. 14b, revealed numerous neurons with high errors (squared error larger than 5), particularly in areas with steep phase changes composed of smaller neuron clusters. In contrast, using λ-sized neurons (middle of Supplementary Figs. 11a-b) improved phase contrast due to more stable effective refractive indices across varying neuron cluster sizes compared to half-λ-sized neurons. For comparison, we also calculated the squared error

distribution in a half-λ-sized-neuron array with a larger Δn of 1.0, shown on the right side of Supplementary Fig. 14b. When plotting the total squared errors (sum of squared errors across all neurons in Supplementary Fig. 14b)[6] against Δn, shown in Supplementary Fig. 14c, the λ-sized-neuron array with a Δn of 0.4 outperformed the half-λ-neuron array with a Δn of 1.0. Such results indicated that adjusting neuron size from half-λ to λ helped minimize the difference between phase modulation calculations and FDTD simulations.

Although the refractive index contrast in our FDTD simulations (Δn = 0.4) differed from the measured refractive index contrast in ImpCarv (Δn = ~0.5, with slightly higher errors compared to Δn = 0.4, shown in Supplementary Fig. 14c), our findings emphasized the importance of considering the intra-array light propagation when designing structures for all-optical machine learning devices.

In summary, we proposed fabricating neurons with a size close to λ to balance the stability of the effective refractive index with the range of diffraction angles. With λ-sized neurons, the phase modulation calculations were close to the FDTD simulations, ensuring the reliability of using phase modulation calculations in the structural design process.

Supplementary Note 3: ImpCarv as a scalable and cost-effective platform for fabricating nanoprecise 3D metastructures

Here we provide the following quantitative assessment based on current capabilities and established pathways for technological advancement.

Regarding scalability, we acknowledge and explicitly state in the manuscript that the point-scanning nature of the two-photon patterning process is the main limitation, as the voxel rate is on the order of 10^6 voxel/sec. By transitioning from point-scanning systems to line-scanning systems, such as spatiotemporal focusing two-photon systems[7], the patterned speed can be increased to 10^9 voxel/sec. The subsequent chemical processing steps, namely ion exchange and supercritical drying, are intrinsically batch processes capable of handling numerous samples simultaneously, which are not limiting steps.

In terms of the processing time and the throughput, let us consider fabricating a nanophotonic device with lateral dimensions of 5×5 mm^2 and comprising 5 layers. Assuming a target voxel size of 50×50×50 nm^3, such a device requires 5×10^{10} voxels in total. Utilizing a parallelized line-scanning photopatterning system with an effective speed of 10^9 voxel/sec, the patterning time for a single device is 50 sec.

For yield, while a formal, high-volume yield analysis is beyond the scope of this foundational method paper, the presented work demonstrates the robustness of the technique. For fabrication precision, the lateral uncertainty is ±10-20 nm and the axial uncertainty is ±2-4 nm. ImpCarv was consistently employed to fabricate complex, multi-layer devices, including the 7-array structure (Supplementary Fig. 16) and the large-area stitched device (Supplementary Fig. 17). Moreover, the consistent device-to-device performance data for the optical machine learning application, obtained from multiple devices across different fabrication batches (Fig. 6a, n=3 devices from two gels for each digit), further highlight the reliability of the method. Initial challenges, such as scaffold cracking during dehydration, which could impact yield, were effectively addressed through the development and implementation of our gradual solvent-exchange protocols.

Finally, we can then consider the cost with the above-mentioned quantitative assessment. The cost are mainly from the material side and the equipment side. For the material side, ImpCarv utilizes readily available and inexpensive materials. Unlike other photocleavable hydrogel methods that may rely on specialized chemistries like nitrobenzyl ether moieties[8,9] or ruthenium polypyridyl complexes[10,11], ImpCarv employs standard, low-cost, commercially sourced chemicals such as hydrogel precursors (*e.g.*, sodium acrylate, acrylamide) and common photosensitizers (*e.g.*, rhodamine B). We estimate the chemical cost to be ~ \$1 per gram of precursor material. Based on an estimated density of the main material in the device[12] (~1.3 g/cm^3), one gram could theoretically produce around 300 devices of the example dimensions (5×5 mm^2 lateral, 0.1 mm axial), resulting in an estimated material cost of only ~\$0.003 per device. For the equipment side, if we assume a typical lifetime for a two-photon laser system (*e.g.*, 8700 hours) and an approximate capital cost (*e.g.*, \$400,000), the operational cost per second is ~\$0.013/sec. Based on the estimated patterning time of 50 sec per device using a parallelized system, the equipment cost contribution would be ~\$0.64 per device. Therefore, the total estimated fabrication cost per device (materials + equipment time) is ~\$0.643. Even accounting for a reasonable process yield (*e.g.*, 75%), the estimated cost remains less than \$1 per device.

In summary, these objective evaluations of potential throughput, processing time, yield characteristics, and component costs strongly support our conclusion that ImpCarv represents a potentially scalable and cost-effective platform for fabricating nanoprecise 3D metastructures.

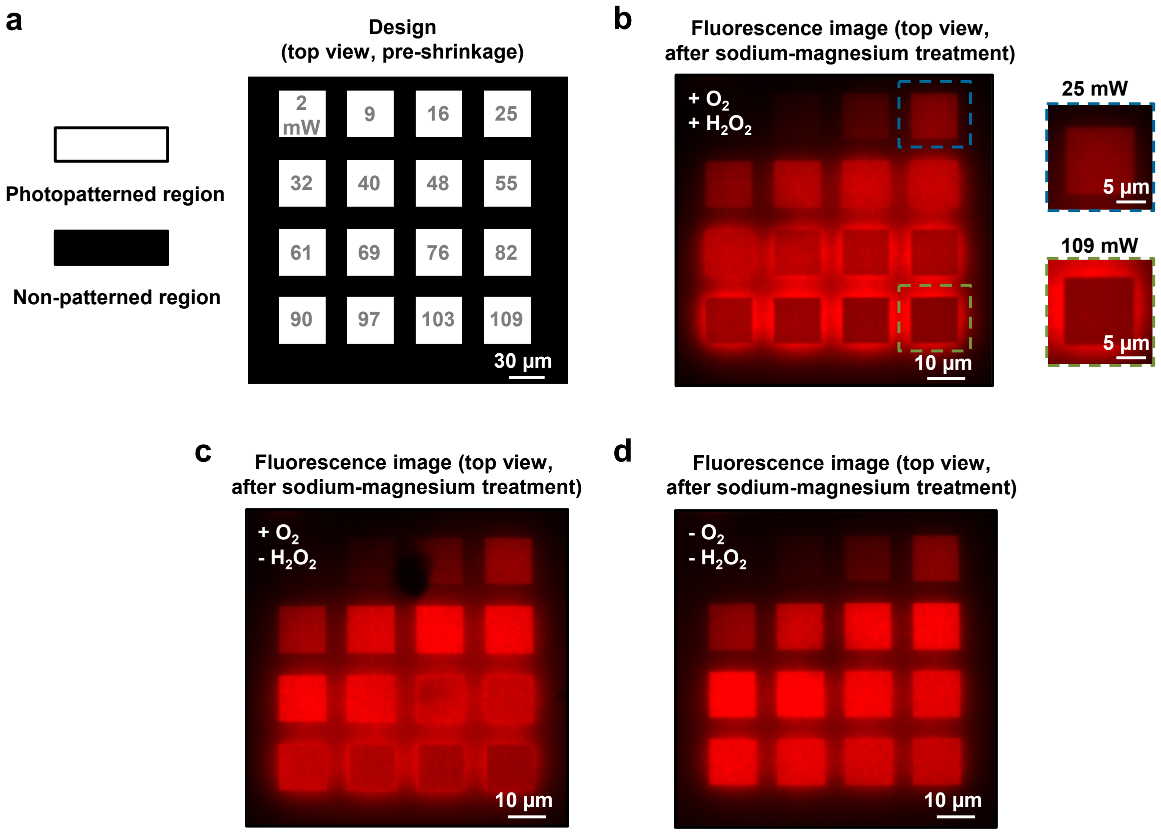

Supplementary Fig. 1: Photopatterning under different laser powers and different conditions of photosensitizer solution. a, Design of a square pattern array with identical dimensions exposed to different laser powers ranging from 2 to 109 mW. White areas indicate photopatterned regions, while black areas represent non-patterned regions. **b**, Single z-slice fluorescence image (from one gel) of the structure described in **a**, using a photosensitizer solution treated with oxygen (O_2) bubbling for 5 min and supplemented with hydrogen peroxide (H_2O_2, concentration: 10 mM), after sodium-magnesium treatment (*i.e.*, 0.02 M NaCl for 15 min followed by 0.1, 0.3, and 0.5 M $MgCl_2$ for 30 min each; details shown in the description of shrinking process in the results) with an approximately three-fold shrinkage factor (MSF hydrogels were used throughout this figure). Insets: magnified views of the structures created with laser powers of 25 mW (top right) and 109 mW (bottom right). **c**, Single z-slice fluorescence image (from one gel) of the structure described in **a**, using a photosensitizer solution treated with O_2 bubbling for 5 min but without H_2O_2, after sodium-magnesium treatment with an approximately three-fold shrinkage factor. **d**, Single z-slice fluorescence image (from one gel) of the structure described in **a**, using a photosensitizer solution without O_2 (O_2 purged by bubbling nitrogen (N_2) for 5 min) or H_2O_2, after sodium-magnesium treatment with an approximately three-fold shrinkage factor. The presence of O_2 and H_2O_2 enhances the efficiency of material cleavage.

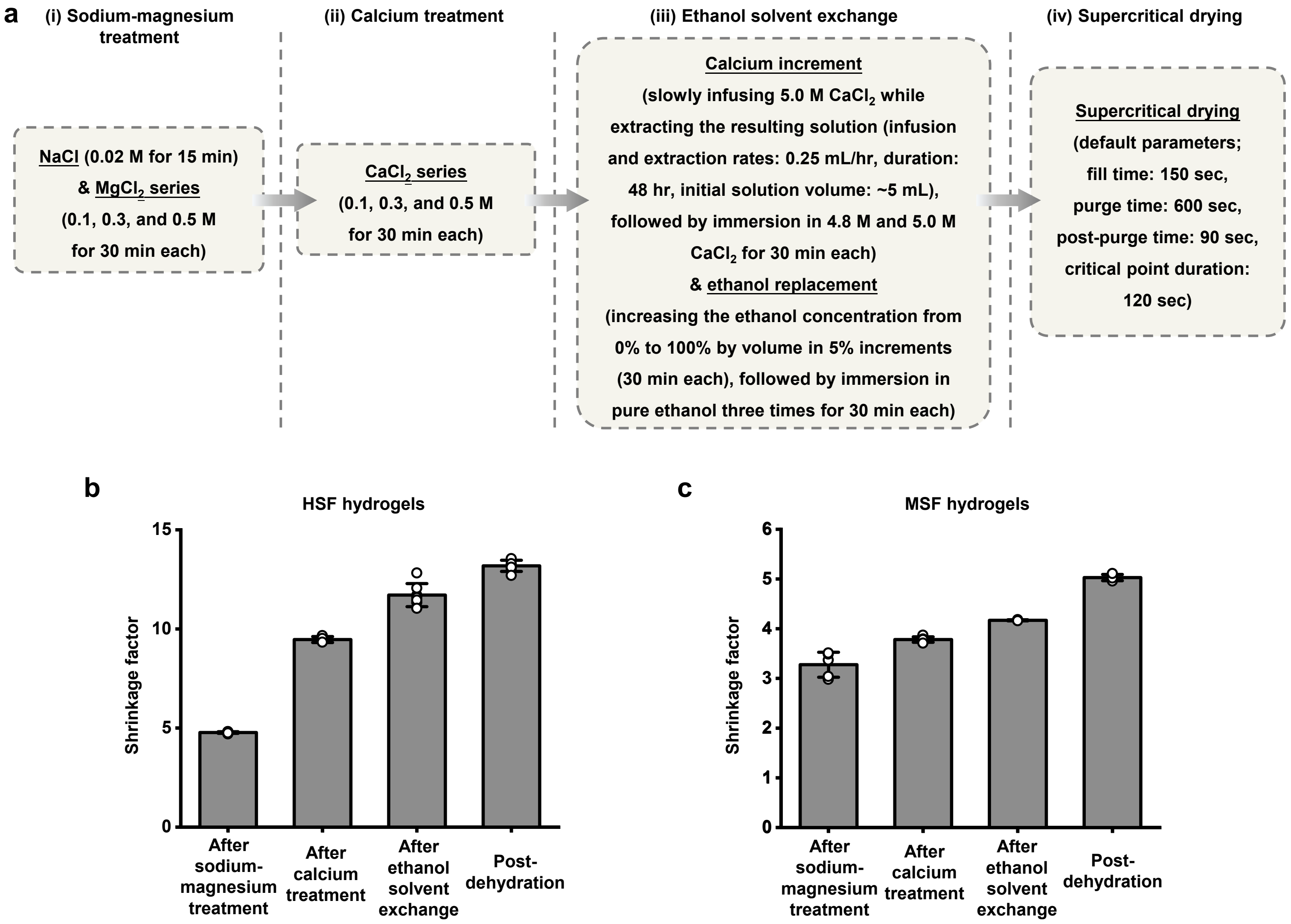

Supplementary Fig. 2: Dehydration process and shrinkage factors for HSF and MSF hydrogels at different stages. a, Dehydration process for both HSF and MSF hydrogels. **b**, Shrinkage factors for HSF hydrogels: 4.78 ± 0.04 (n = 7 areas from three gels; open circular points, bars, and error bars represent individual data points, means, and SD, respectively, used throughout this paper) after sodium-magnesium treatment, 9.47 ± 0.15 (n = 4 areas from two gels) after calcium treatment, 11.71 ± 0.58 (n = 7 areas from three gels) after ethanol solvent exchange, and 13.18 ± 0.28 (n = 7 areas from four gels) post-dehydration. **c**, Shrinkage factors for MSF hydrogels: 3.28 ± 0.25 (n = 5 areas from five gels; open circular points, bars, and error bars represent individual data points, means, and SD, respectively, used throughout this paper) after sodium-magnesium treatment, 3.78 ± 0.06 (n = 7 areas from seven gels) after calcium treatment, 4.17 ± 0.01 (n = 3 areas from three gels) after ethanol solvent exchange, and 5.03 ± 0.06 (n = 5 areas from five gels) post-dehydration.

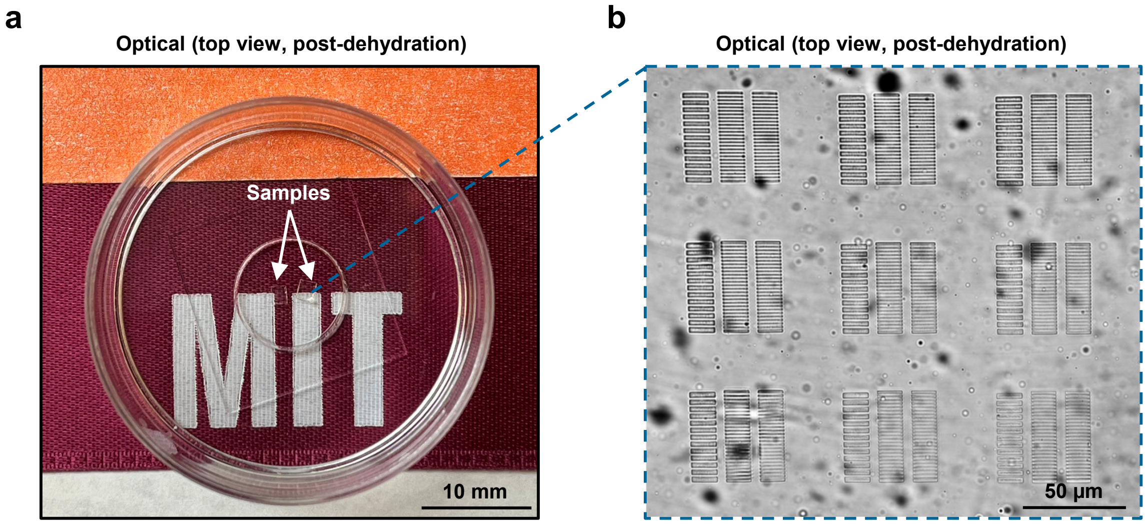

Supplementary Fig. 3: Appearance of samples post-dehydration. a, Samples remain transparent post-dehydration (HSF hydrogels were used throughout this figure; with the 'MIT' letter pattern serving as a background to highlight the transparency of samples). **b**, Optical image (from one gel) showing that internal structures exhibit a distinct intensity contrast relative to the scaffold under an optical microscope. The structures visible in the optical image are gratings with different depths.

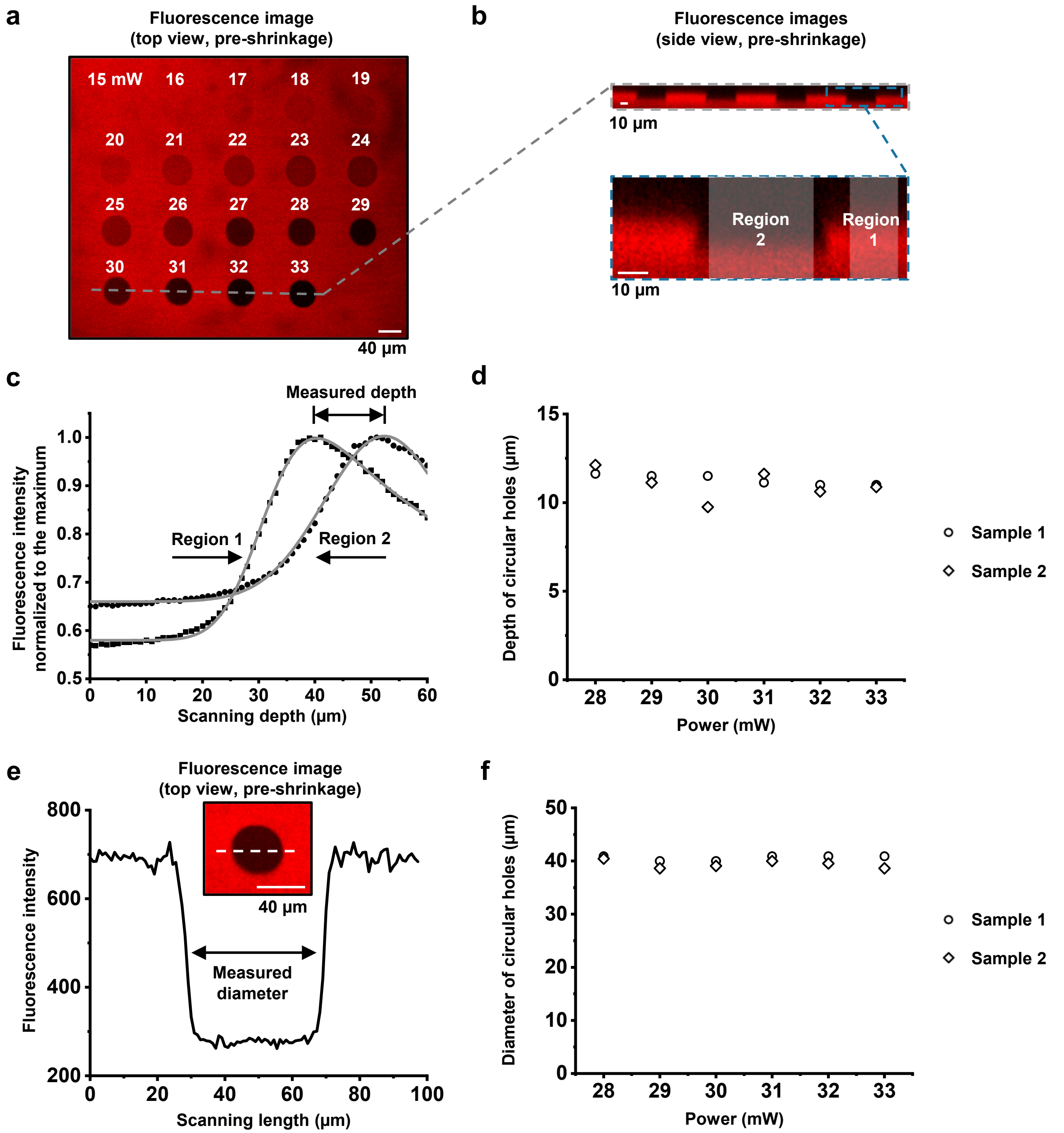

Supplementary Fig. 4: Geometry measurements of circular holes based on fluorescence intensity analysis. a, Representative single z-slice fluorescence image (n = 2 arrays from two gels) of the structure described in Fig. 2a in swollen hydrogel (HSF hydrogels were used throughout this figure). **b**, Side view fluorescence image obtained by z-stacking fluorescence images along the gray dashed line in **a**. Blue inset: magnified view of the blue dashed frame in **b**, highlighting regions 1 and 2 for depth measurements. **c**, Measured fluorescence intensity (points), normalized to the maximum, as a function of scanning depth for regions 1 and 2 in the blue inset of **b**. Lines represent fitting curves based on a double Gaussian distribution. The distance between the two peaks of these curves determines the depth of the circular hole. **d**, Depths of the circular holes in the two swollen hydrogels, treated with laser powers of 28 mW or higher, used to generate Fig. 2b (open circular points and open rhombic points represent individual data points from gel 1 and gel 2, respectively, used throughout this figure). **e**, Measured fluorescence intensity profile along the white dashed line in the inset. The full width at half maximum (FWHM) value is used to determine the diameter of a circular hole. Inset: single z-slice fluorescence image of a representative circular hole in swollen hydrogel. **f**, Diameters of the circular holes in the two swollen hydrogels, treated with laser powers of 28 mW or higher, used to generate Fig. 2b.

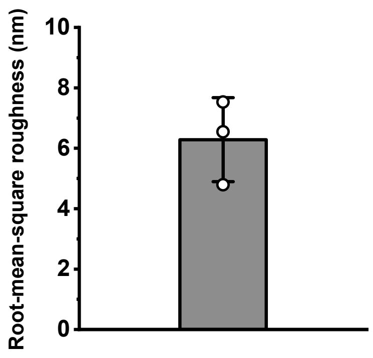

Supplementary Fig. 5: Root-mean-square roughness of the surface in photopatterned regions with a laser power above threshold (*i.e.*, 30 mW) measured by AFM. The surface roughness is 6.3 ± 1.4 nm post-dehydration (n = 3 areas from three gels; open circular points, bars, and error bars represent individual data points, means, and SD, respectively; MSF hydrogels were used in this figure).

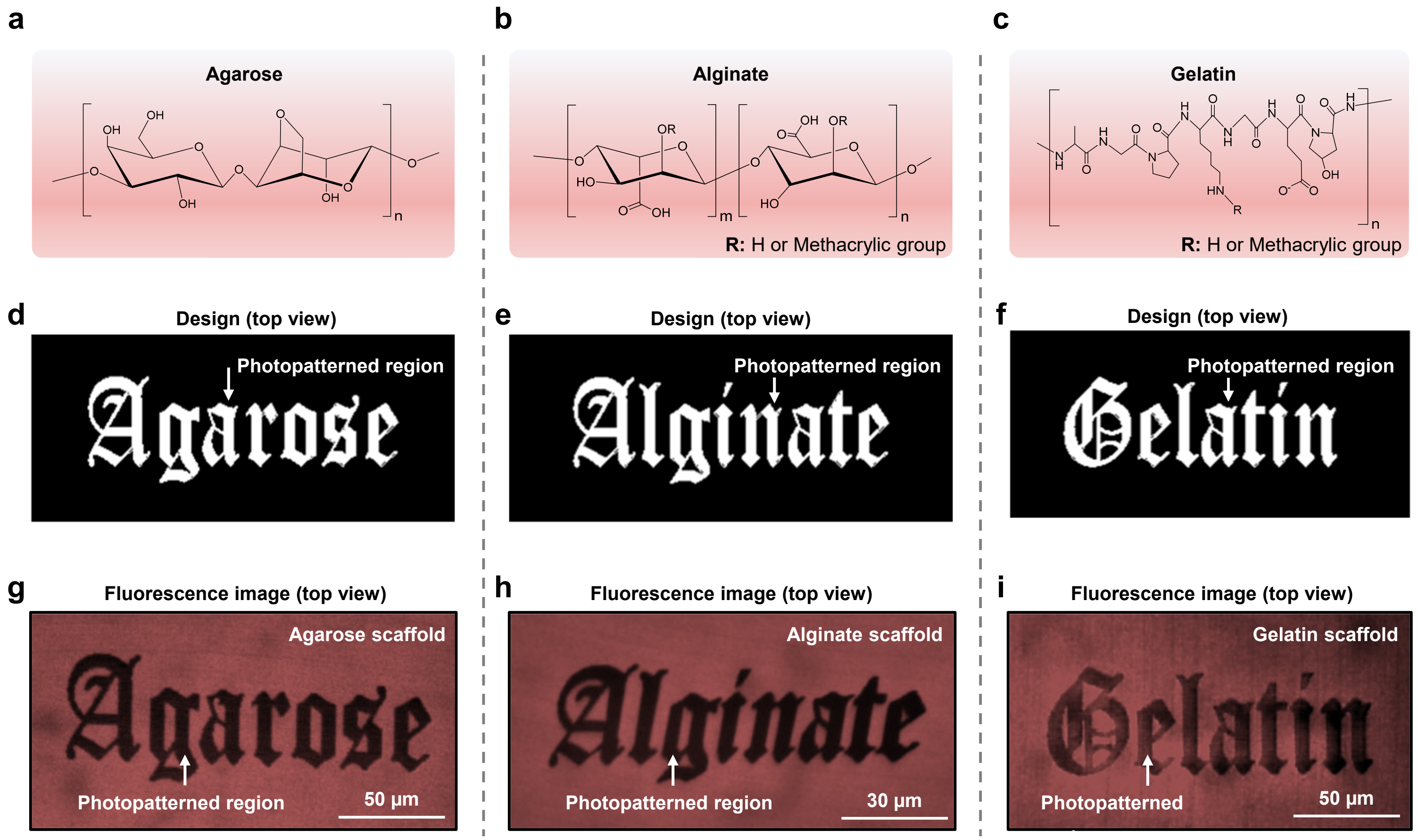

Supplementary Fig. 6: Versatility for diverse hydrogel scaffolds. a-c, Chemical structures of several hydrogels used in this experiment including agarose (**a**), alginate (**b**), and gelatin (**c**) hydrogels. **d-f**, Design of test patterns for these hydrogels, with white areas representing photopatterned regions and grey areas indicating non-patterned regions. The patterns correspond to the shapes of 'Agarose' (**d**), 'Alginate' (**e**), and 'Gelatin' (**f**) for these hydrogels, respectively. These patterns were generated within the hydrogel scaffold, followed by cleavage of the hydrogel above the design pattern. Initial designs aimed to enable SEM or AFM imaging of the patterns by cleaving above the scaffold; however, due to the anisotropic hydrogel deformation during the ethanol solvent exchange, it was not feasible to dehydrate these gels while preserving the structures. As a result, fluorescence imaging in an aqueous solution (*e.g.*, PBS (1×)) was employed instead. **g-i**, Representative sum-intensity projections of z-stacked fluorescence images (n = 2 test patterns from two gels per gel type) of the structures described in **d-f** within agarose (**g**), alginate (**h**), and gelatin (**i**) hydrogels, respectively. Fluorescence images were captured after immersing the hydrogels in PBS (1×) solution, where alginate hydrogels showed a modest shrinkage factor of ~1-2, while the other hydrogels did not exhibit noticeable shrinkage. The fluorescence contrast in the gelatin hydrogels was slightly weaker compared to the other hydrogels, likely due to the lower density of photosensitizers anchored at the boundaries of the photopatterned regions.

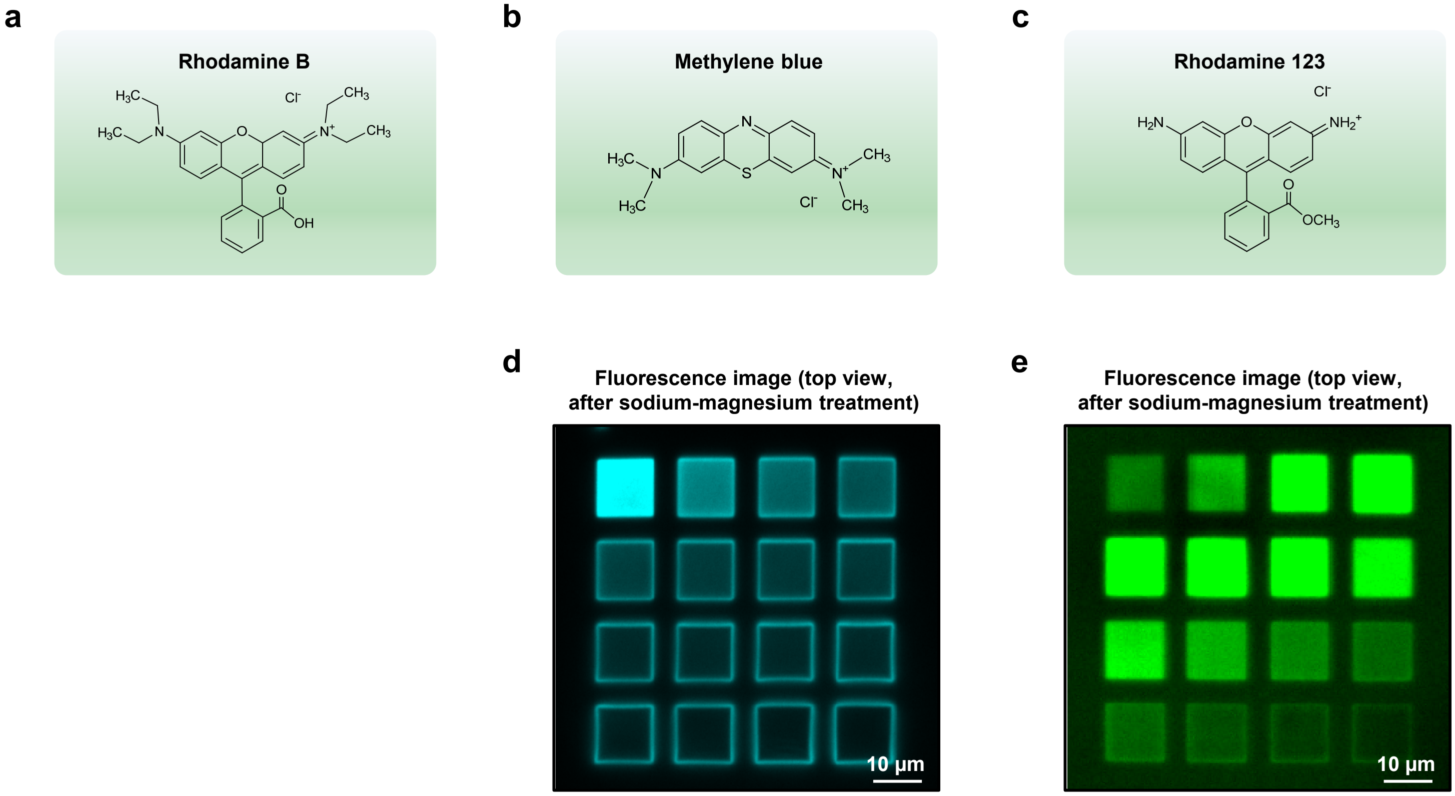

Supplementary Fig. 7: Versatility for diverse photosensitizers. a-c, Photosensitizers utilized in this experiment, including rhodamine B (**a**), methyl blue (**b**), and rhodamine 123 (**c**). Design of the test pattern appears in Supplementary Fig. 1a, and the results using rhodamine B are presented in Supplementary Fig. 1b. **d-e**, Single z-slice fluorescence images of the structure (each from one gel) described in Supplementary Fig. 1a, using methylene blue (**d**) and rhodamine 123 (**e**), after sodium-magnesium treatment with an approximately three-fold shrinkage factor (MSF hydrogels were used throughout this figure). Consistent with Supplementary Fig. 1b, the aqueous photosensitizer solutions in **d-e** were treated with O_2 bubbling for 5 min and supplemented with H_2O_2 (concentration: 10 mM).

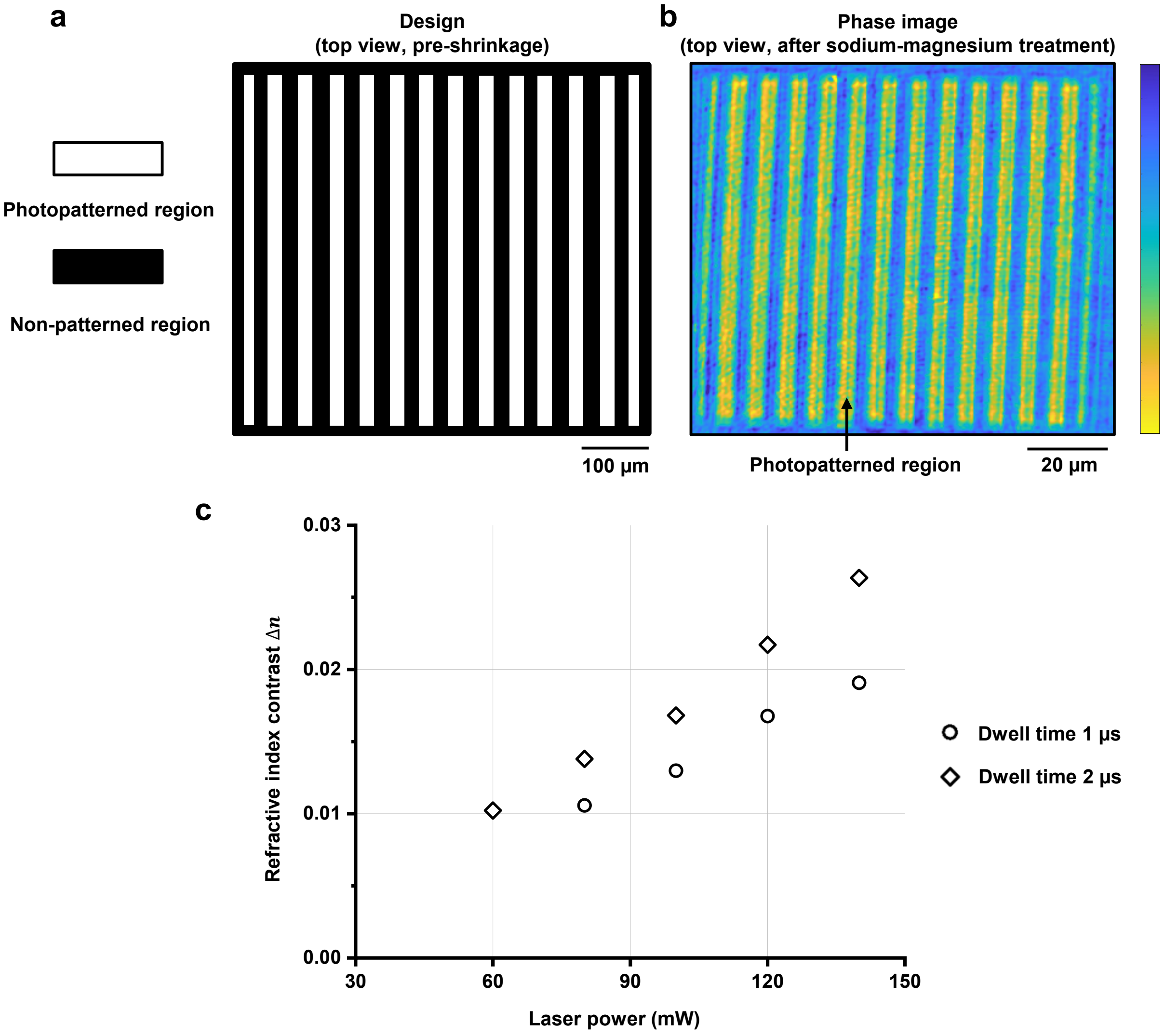

Supplementary Fig. 8: Refractive index contrast (Δn) after sodium-magnesium treatment. **a**, Design of the test pattern at a depth of 20 μm, with white areas representing photopatterned regions and black areas indicating non-patterned regions. **b**, Phase image (from one gel) of the structure described in **a** after sodium-magnesium treatment with an approximately five-fold shrinkage factor (HSF hydrogels were used throughout this figure; laser power: 140 mW, dwell time: 2 μs). The Δn is ~0.026 between photopatterned vacancies and non-patterned regions, calculated using the equation $\Delta\varphi = (2\pi \times \Delta n \times h)/\lambda$, where h represents the depth of photopatterned vacancies and and λ is the wavelength of light (633 nm). Here, h was obtained by dividing the depth of the test pattern by the shrinkage factor (~5); the focal depth of the laser in the two-photon system (~2 μm) was neglected because it was smaller than the depth of the test pattern. **c**, Variation in Δn (each Δn from one gel) as a function of photopatterning parameters (laser power and dwell time), showcasing a refractive index contrast gradient achievable by controlling the percentage of hydrogel material cleavage in photopatterned regions. Open circular points and open rhombic points represent individual data points under a dwell time of 1 μs and 2 μs, respectively.

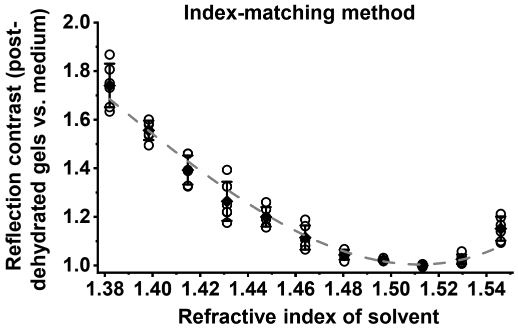

Supplementary Fig. 9: Index-matching method to examine the refractive index of the post-dehydrated gels. The index-matching method uses mixtures of hexane (refractive index: 1.382 at 460 nm) and benzyl ether (refractive index: 1.546 at 460 nm). The post-dehydrated gels were immersed in the mixed medium, and the reflection contrast between the gel and the surrounding medium was observed under a microscope while varying the solvent composition (n = 6 measurements from three gels; open circular points, solid rhombic points, and error bars represent individual data points, means, and SD, respectively; gray dashed line refers to the quadratic fitting; MSE hydrogels were used in this figure). The results indicate that the refractive index of the post-dehydrated gels is ~1.5.

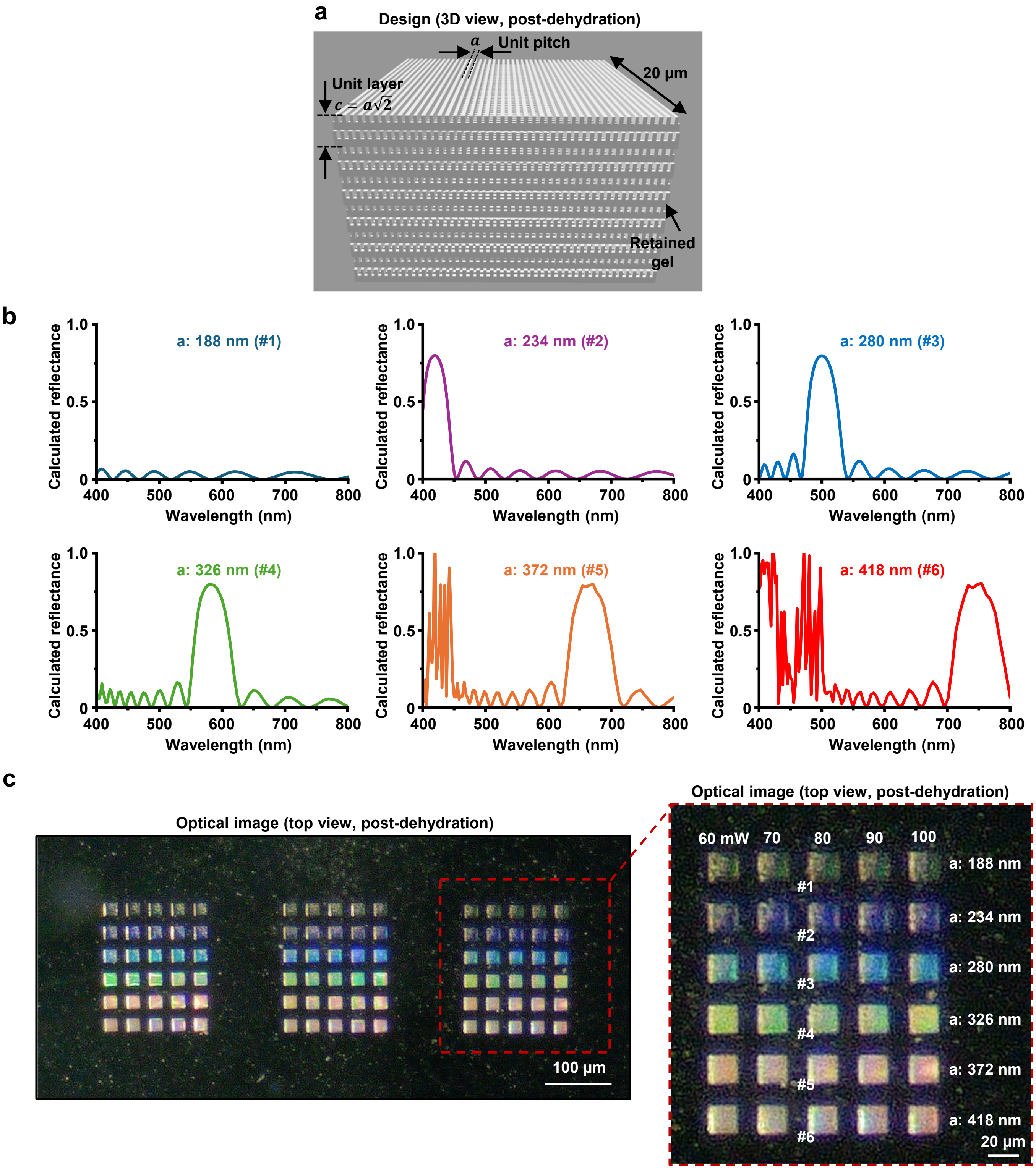

a
Design (3D view, post-dehydration)
a
Unit pitch
20 µm
Unit layer
$c = a\sqrt{2}$
Retained
gel
b
a: 188 nm (#1)
a: 234 nm (#2)
a: 280 nm (#3)
a: 326 nm (#4)
a: 372 nm (#5)
a: 418 nm (#6)
Calculated reflectance
Wavelength (nm)
c
Optical image (top view, post-dehydration)
100 µm
Optical image (top view, post-dehydration)
60 mW
70
80
90
100
a: 188 nm
a: 234 nm
a: 280 nm
a: 326 nm
a: 372 nm
a: 418 nm
#1
#2
#3
#4
#5
#6
20 µm

Supplementary Fig. 10: 3D photonic crystals with nanoprecise structures indicating a photonic bandgap in the visible spectral range. a, Design of a 3D photonic crystal woodpile structure within the hydrogel (the dimensions labeled are for post-dehydrated status), with edges connected to the hydrogel scaffold. The unit pitch and layer, a and c ($c = a\sqrt{2}$), refers to dimensions of the lateral and axial repeating units, respectively. The 3D photonic crystal structure has 6 layers and a footprint of 20×20 μm^2. **b**, Calculated reflectance spectra for 3D photonic crystals based on different unit pitch values (dark teal, purple, blue, green, orange, red for 188, 234, 280, 326, 372, 418 nm, respectively), using finite-difference time-domain (FDTD) method. **c**, Representative true-color reflection-mode optical image of the structure described in **a** post-dehydration (n = 6 arrays from two gels; each array contains 30 photonic crystal structures with different values for unit pitch and layer, and with different photopatterning powers; MSF gels were used throughout this figure). Red inset: visible colors are obvious in the 3D photonic crystal structures, with a unit pitch of 234 nm for #2, 280 nm for #3, 326 nm for #4, 372 nm for #5, and 418 nm for #6. The results show that the 3D photonic crystals, fabricated by ImpCarv, indicate a photonic bandgap in the visible spectral range.

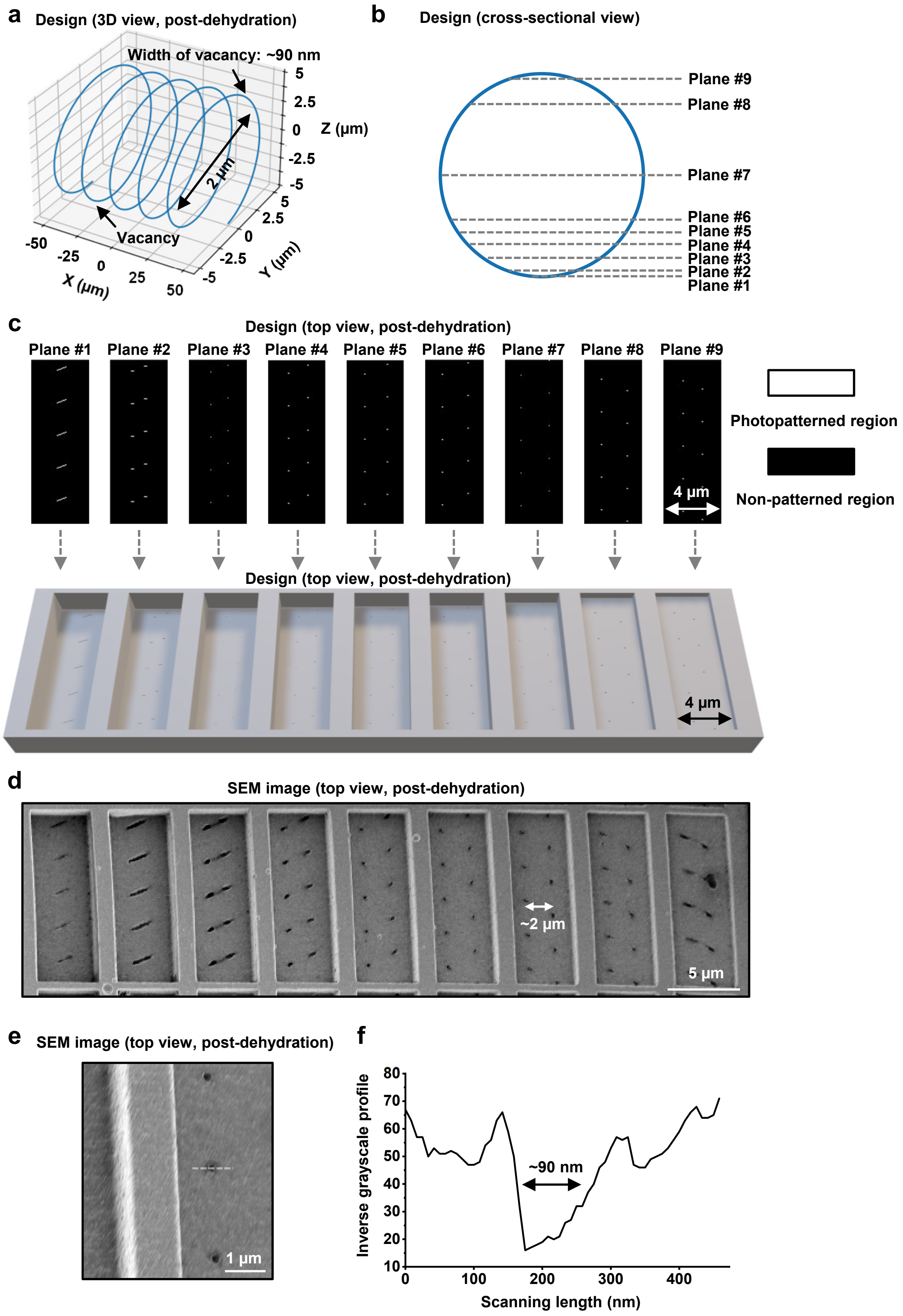

a
Design (3D view, post-dehydration)
Width of vacancy: ~90 nm
2 μm
Vacancy
X (μm)
Y (μm)
Z (μm)
b
Design (cross-sectional view)
Plane #9
Plane #8
Plane #7
Plane #6
Plane #5
Plane #4
Plane #3
Plane #2
Plane #1
c
Design (top view, post-dehydration)
Photopatterned region
Non-patterned region
4 μm
Design (top view, post-dehydration)
4 μm
d
SEM image (top view, post-dehydration)
~2 μm
5 μm
e
SEM image (top view, post-dehydration)
1 μm
f
~90 nm
Inverse grayscale profile
Scanning length (nm)

Supplementary Fig. 11: 3D vacant spirals with nanoprecise structures. a, Design of a 3D vacant spiral structure within the hydrogel (the dimensions labeled are for post-dehydration status). **b**, For easy imaging, multiple 3D vacant spiral structures are created in a row, and then exposed to imaging at nine different planes (#1-9). **c**, Top row: design of each plane from #1-9, with white areas representing photopatterned regions and black areas indicating non-patterned regions. Bottom row: layout of these 3D vacant spiral structures with different exposed planes from #1-9. The dimensions labeled are for post-dehydration status. **d**, Representative SEM image of the structure described in **c** post-dehydration (n = 8 structures from two gels; HSF gels were used throughout this figure). The image shows that the 3D vacant spiral has a diameter of ~ 2 µm. **e**, Representative SEM image of the spiral width in the structure described in **c** post-dehydration (n = 8 structures from two gels). **f**, Cross-section inverse grayscale profile of the spiral width along the dashed line in **e** indicates that the 3D vacant spiral has a width of ~90 nm.

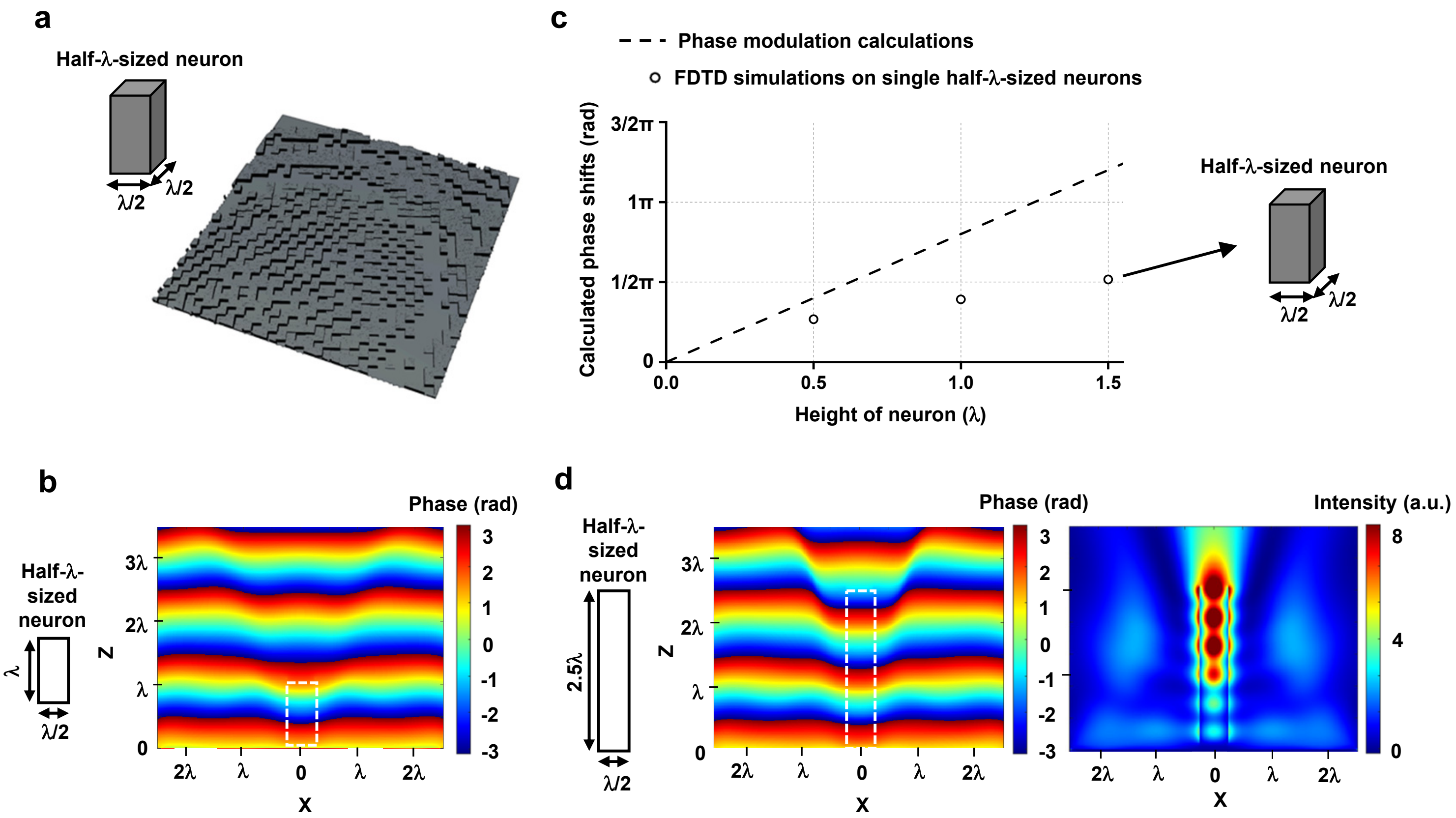

Supplementary Fig. 12: Phase distribution and shifts in half-λ-sized neurons. a, Schematic of the array design for all-optical machine learning devices using half-λ-sized neurons. **b**, Cross-sectional view of a half-λ-sized neuron with a height of λ (left) and its corresponding phase distribution (right), obtained via FDTD simulations. In this setup, a linearly polarized plane wave (polarized along the x-direction) enters from below and propagates through the neuron. Phase modulation calculations indicated a phase shift of 0.8π for this neuron, whereas FDTD simulations indicated a phase shift of only 0.4π. **c**, Phase shifts as a function of neuron height. Open circular points represent individual FDTD simulation results for single half-λ-sized neurons with varying heights, while the black dashed line corresponds to results based on phase modulation calculations. For these neurons, phase shifts obtained from FDTD simulations were considerably lower than those obtained by phase modulation calculations. **d**, Cross-sectional view of a half-λ-sized neuron with a height of 2.5λ (left), its phase distribution (middle), and its intensity distribution (right), all obtained via FDTD simulations. All calculation parameters are consistent with **b**, except for the neuron height. In this case, phase modulation calculations indicated a 2π phase shift; however, only a 0.64π phase shift was observed according to FDTD simulations.

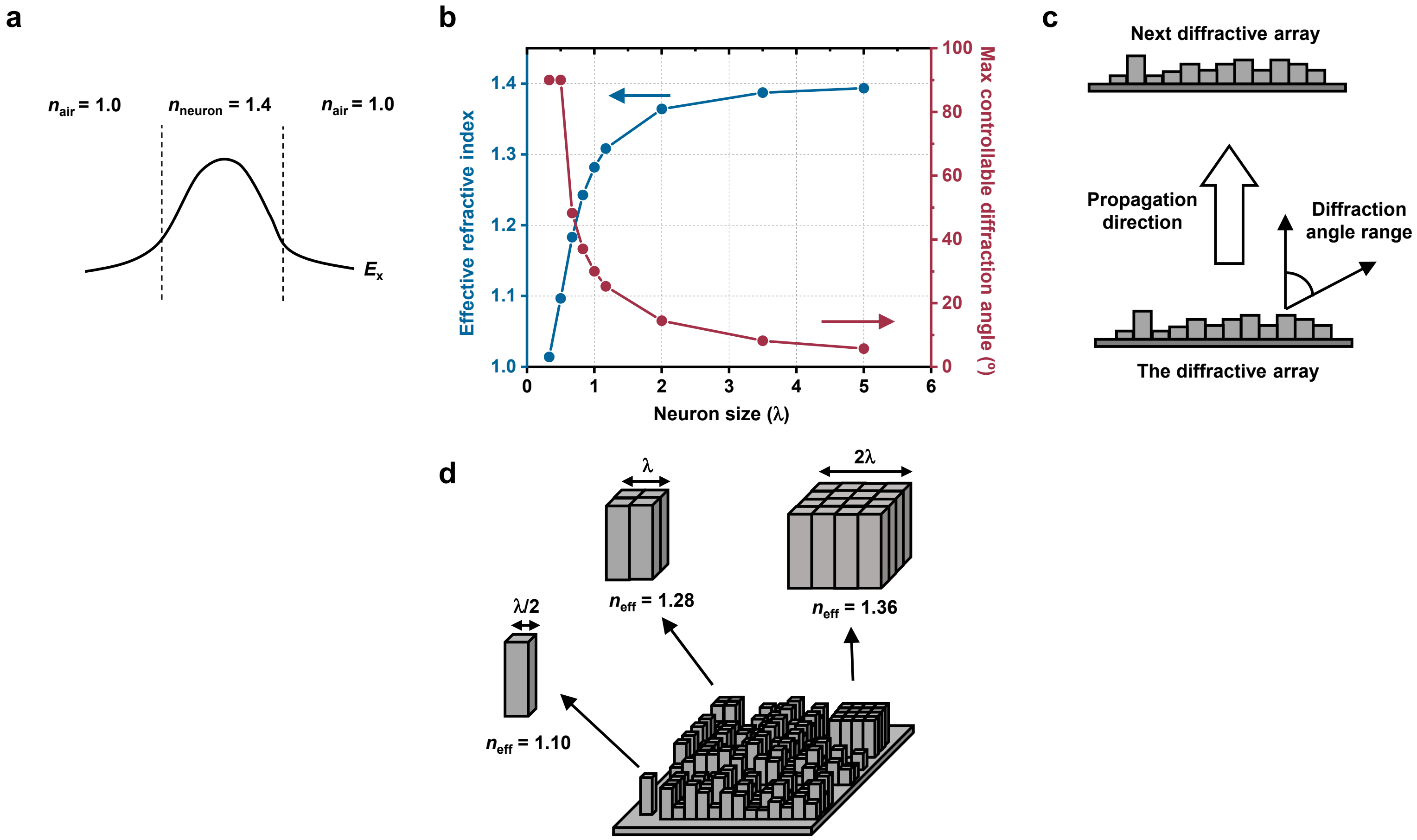

Supplementary Fig. 13: Impact of neuron size on refractive indices and diffraction angles. a, Schematic illustrating that due to the distribution of the electric field of light within both the neuron and the surrounding medium, the effective refractive index for tiny neurons tends to be smaller than refractive index of the material itself. **b**, Effective refractive indices (blue points) and maximum controllable diffraction angle (red points) as functions of neuron sizes. **c**, Schematic depicting the diffraction angle range achievable within a single diffractive array. **d**, Schematic of a neuron array, showing numerous neurons organized into clusters of varying sizes. Each cluster exhibits a distinct effective refractive index that depends on the size of the neuron clusters.

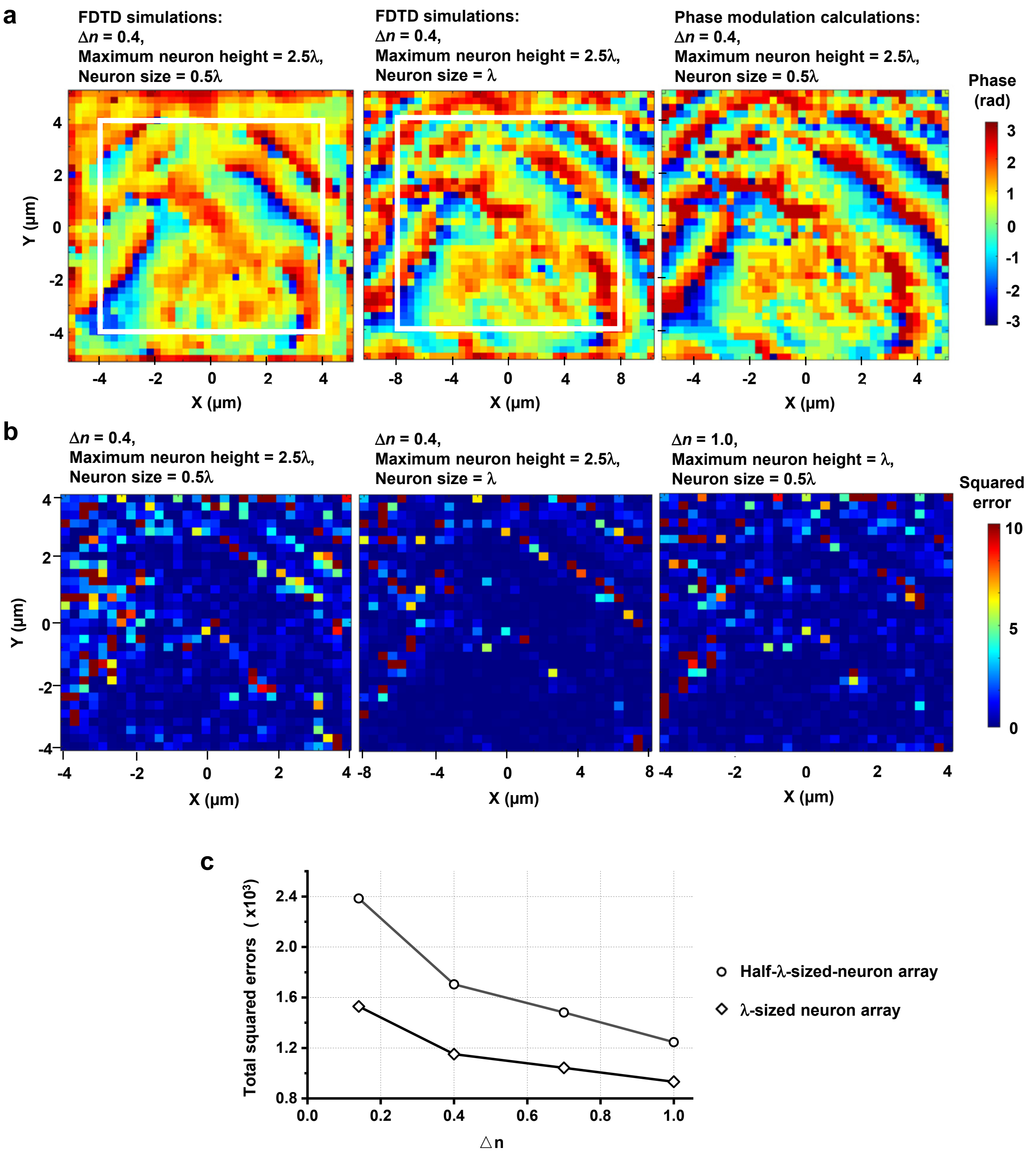

Supplementary Fig. 14: Phase distributions and error analysis. a, Phase distribution of a 30×30 neuron array under different conditions, using FDTD simulations and phase modulation calculations. The left panel shows the phase distribution for a half-λ-sized-neuron array ($\Delta n = 0.4$) obtained through FDTD simulations; the middle panel depicts the results for a λ-sized-neuron array ($\Delta n = 0.4$) using FDTD simulations, while the right panel presents the phase distribution for a half-λ-sized-neuron array ($\Delta n = 0.4$) based on phase modulation calculations. The incident light is a plane wave, linearly polarized along the x-direction and propagating in the z-direction. **b**, Squared error at each neuron, determined by squaring the phase difference between the phase modulation calculations and the FDTD simulations. The left panel depicts the results for a half-λ-sized-neuron array ($\Delta n = 0.4$), the middle panel for a λ-sized-neuron array ($\Delta n = 0.4$), and the right panel for a half-λ-sized-neuron array ($\Delta n = 1.0$). The error analysis focuses on the inner region (indicated by the white frame in **a**) to minimize the edge diffraction effects. **c**, Total squared error as a function of refractive indices, calculated by summing the squared errors across all neurons in **b**. Open circular points and open rhombic points represent individual results for half-λ-sized-neuron and λ-sized-neuron arrays, respectively.

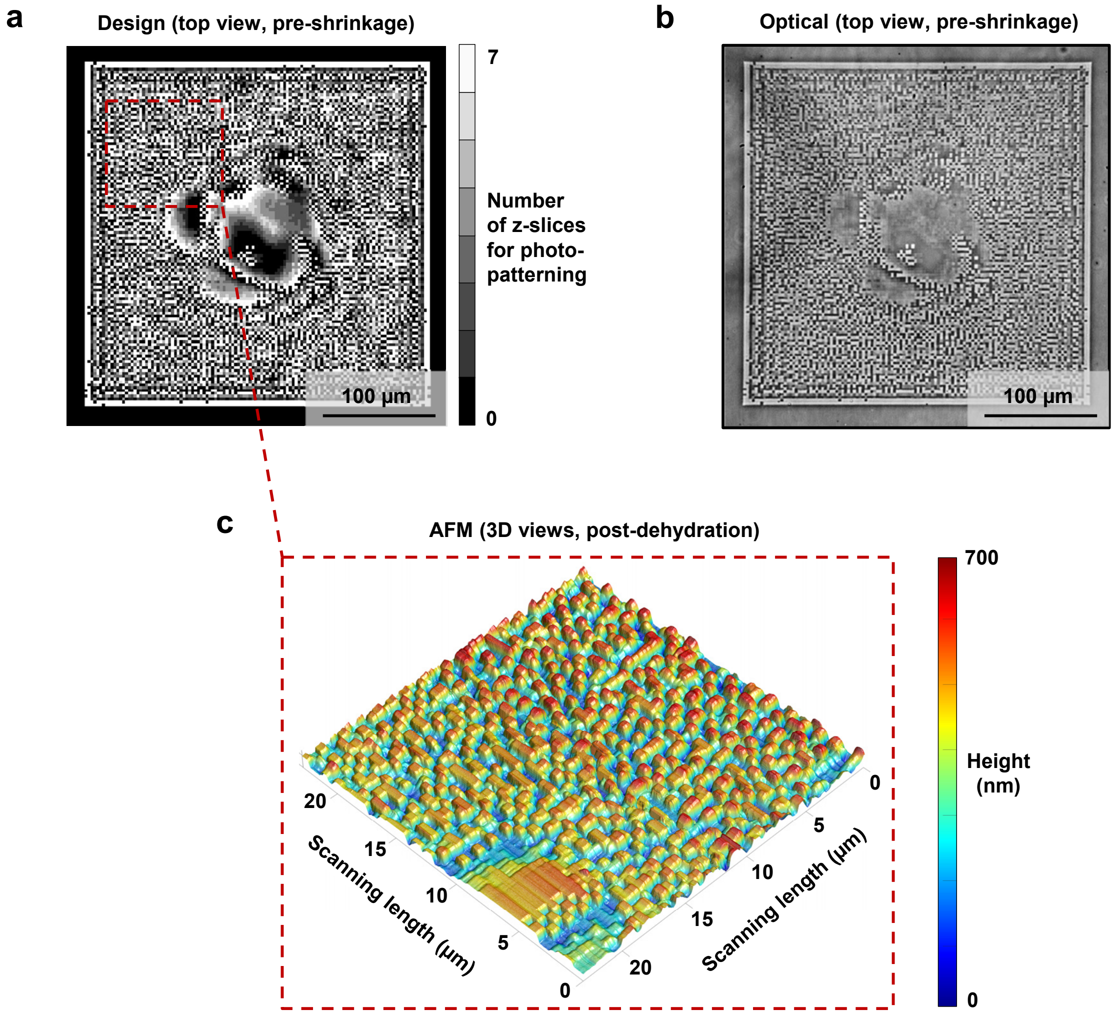

Supplementary Fig. 15: Additional AFM measurement on the device structures. a, Design of a single array of the device. The fabrication of the array with eight discrete heights was achieved by photopatterning across seven z-slices (step size between consecutive slices: 0.5 µm). **b**, Representative optical image (n = 2 arrays from two gels) of the structure described in **a** in swollen hydrogel (MSF hydrogels were used throughout this figure). **c**, Representative 3D AFM height profile of the structure from the red dashed frame in **a** post-dehydration (n = 2 areas from two arrays from two gels; AFM scanning area: ~23 × 23 µm), corresponding to the experimental results in Fig. 5e.

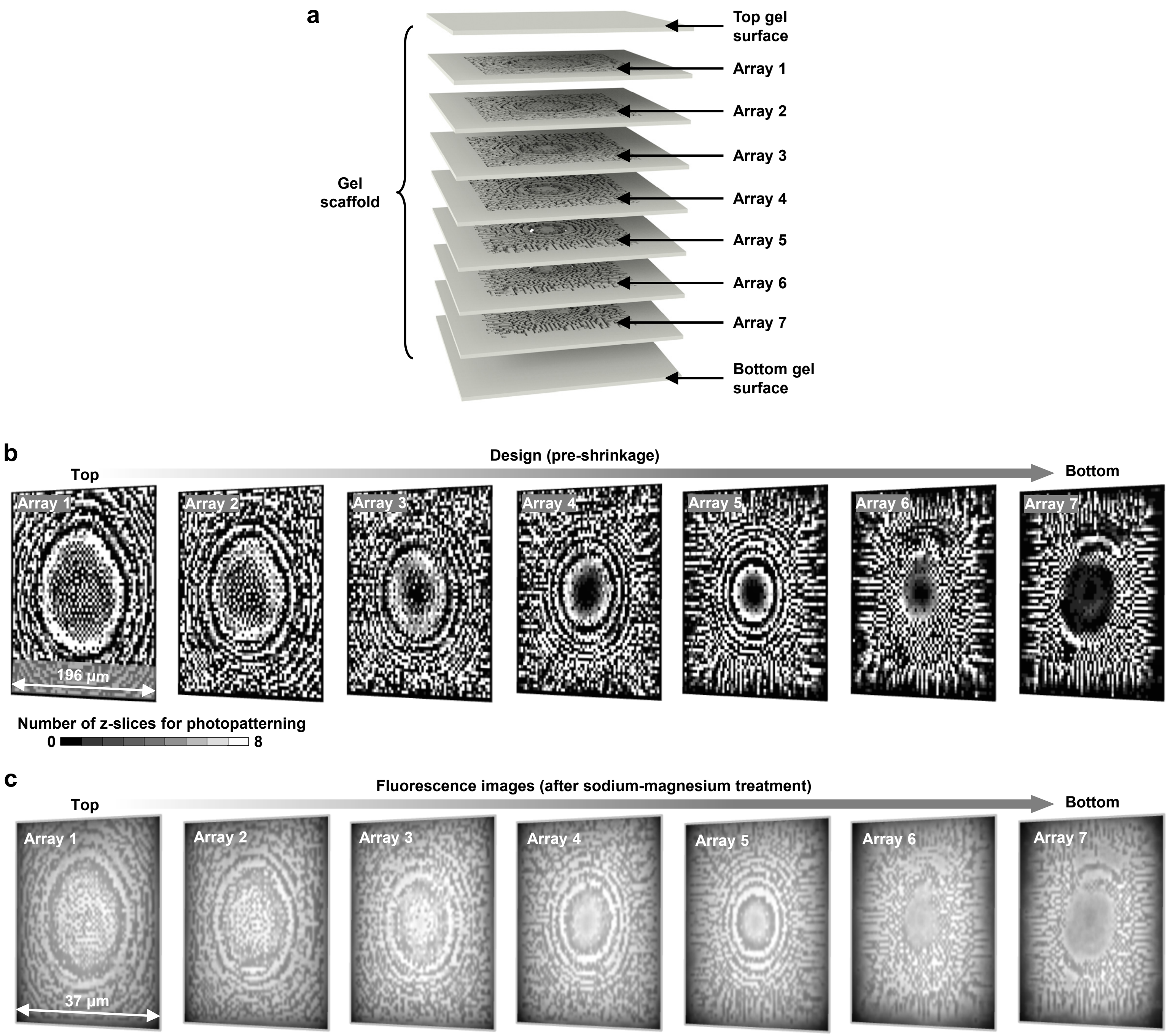

Supplementary Fig. 16: Seven-array 3D metastructures. **a**, Design of the seven-array 3D metastructures, each array featuring neurons with multiple height steps. **b**, Design of each array in the 3D metastructure. The fabrication of each array, with varied heights, was achieved by photopatterning eight z-slices (step size between consecutive slices: 1.7 µm), with each slice retaining a different fraction of the polymer. The hydrogel material between adjacent arrays, with a pre-shrink thickness of ~15 µm, provided mechanical stability to the structure. **c**, Representative sum-intensity projections of z-stacked fluorescence images (n = 16 structures from two gels) for each array of the structure described in **a-b** after sodium-magnesium treatment with an approximately five-fold shrinkage factor.

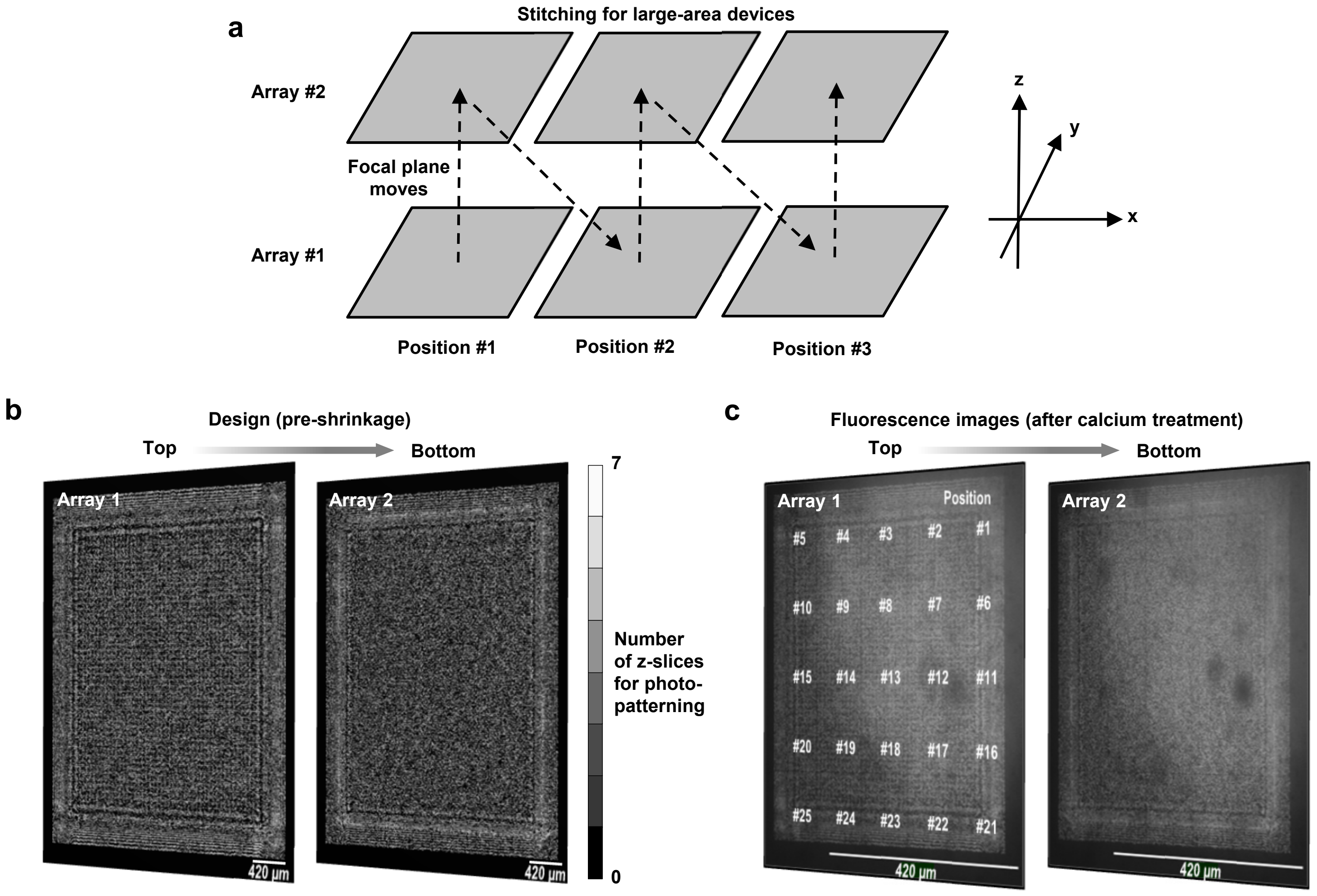

Supplementary Fig. 17: Large-area devices with half-millimeter-level overall dimensions achieved by stitching multiple photopatterned regions. a, Illustration of the stitching process across multiple lateral positions and axial arrays. **b**, Design of a large-area device composed of two arrays, each containing 420 × 420 neurons. The fabrication of each array with eight discrete heights was achieved by photopatterning across seven z-slices (step size between consecutive slices: 1.0 μm). **c**, Sum-intensity projections of z-stacked fluorescence images (from one gel) of each array after calcium treatment with an approximately four-fold shrinkage factor (MSF hydrogels were used in this figure). The device achieved a lateral dimension of 525 × 525 μm by stitching 25 photopatterned regions.

Supplementary Table 1: Formulation of the HSF hydrogel

Solution number	Components	Comment
Solution A	Sodium acrylate (26.0 w/v%), acrylamide (7.5 w/v%), and PBS (10×, 16.7 vol%) in UltraPure water	None
Solution B	MBAA (2.0 w/v%) in UltraPure water	None
Solution C	APS (10.0 w/v%) in UltraPure water	Use immediately after preparation
Solution D	TEMED (10.0 vol%) in UltraPure water	Use immediately after preparation
Solution E	600 µL solution A + 13 µL solution B + 347 µL UltraPure water	After mixing, N_2 bubbling for 3 min
Final precursor solution	192 µL solution E + 4 µL solution D + 4 µL solution C in sequence	Cast the solution into a hydrophobic mold and keep the samples in 37 °C oven for at least 3 hr for gelation

Supplementary Table 2: Formulation of the MSF hydrogel

Solution number	Components	Comment
Solution A	Sodium acrylate (39.0 w/v%), acrylamide (11.3 w/v%), and PBS (10×, 16.7 vol%) in UltraPure water	None
Solution B	MBAA (2.0 w/v%) in UltraPure water	None
Solution C	APS (10.0 w/v%) in UltraPure water	Use immediately after preparation
Solution D	TEMED (10.0 vol%) in UltraPure water	Use immediately after preparation
Solution E	600 µL solution A + 120 µL solution B + 240 µL UltraPure water	After mixing, N_2 bubbling for 3 min
Final precursor solution	192 µL solution E + 4 µL solution D + 4 µL solution C in sequence	Cast the solution into a hydrophobic mold and keep the samples in 37 °C oven for at least 3 hr for gelation

Supplementary Table 3: Summary of experimental procedures used to generate each figure

Sample	Hydrogel scaffold	Photosensitizer solution	Photopatterning parameters	Shrinking process	Ethanol solvent exchange	Supercritical drying
2b, S4	HSF hydrogel	150 µM rhodamine B, 10 mM H_2O_2, isopropylamine added to tune pH to ~ 8.0-9.5, O_2 bubbling for 5 min	15-33 mW power, 6 µs dwell time, 1 µm step size, 20× objective	None	None	None
2c	HSF hydrogel	150 µM rhodamine B, 10 mM H_2O_2, isopropylamine added to tune pH to ~ 8.0-9.5, O_2 bubbling for 5 min	15-33 mW power, 6 µs dwell time, 1 µm step size, 20× objective	NaCl (0.02 M for 15 min) → $MgCl_2$ (0.1, 0.3, and 0.5 M for 30 min each) → $CaCl_2$ (0.1, 0.3, and 0.5 M for 30 min each)	Yes	Yes
2e, 2g	HSF hydrogel	150 µM rhodamine B, 10 mM H_2O_2, isopropylamine added to tune pH to ~ 8.0-9.5, O_2 bubbling for 5 min	30 mW power, 6 µs dwell time, 1 µm step size, 20× objective	NaCl (1.0 M)	None	None
3b, 3d, 3e, 3f, 3h, 3i, 3j, S3, S5	HSF hydrogel	150 µM rhodamine B, 10 mM H_2O_2, isopropylamine added to tune pH to ~ 8.0-9.5, O_2 bubbling for 5 min	30 mW power, 6 µs dwell time, 250 nm step size, 20× objective	NaCl (0.02 M for 15 min) → $MgCl_2$ (0.1, 0.3, and 0.5 M for 30 min each) → $CaCl_2$ (0.1, 0.3, and 0.5 M for 30 min each)	Yes	Yes
3c, 3g	HSF hydrogel	150 µM rhodamine B, 10 mM H_2O_2, isopropylamine added to tune pH to ~ 8.0-9.5, O_2 bubbling for 5 min	30 mW power, 6 µs dwell time, 250 nm step size, 20× objective	None	None	None
4b, 4c, 4d	MSF hydrogel	250 µM rhodamine B, 10 mM H_2O_2, isopropylamine added to tune pH to ~ 8.0-9.5, O_2 bubbling for 5 min	90 and 100 mW power, 4 µs dwell time, 0.75 and 1.0 µm step size, 20× objective	NaCl (0.02 M for 15 min) → $MgCl_2$ (0.1, 0.3, and 0.5 M for 30 min each) → $CaCl_2$ (0.1, 0.3, and 0.5 M for 30 min each)	Yes	Yes
5c	MSF hydrogel	250 µM rhodamine B, 10 mM H_2O_2, isopropylamine added to tune pH to ~ 8.0-9.5, O_2 bubbling for 5 min	80 mW power, 3 µs dwell time, 0.5 µm step size, 20× objective	None	None	None
5d	MSF hydrogel	250 µM rhodamine B, 10 mM H_2O_2, isopropylamine added to tune pH to ~ 8.0-9.5, O_2 bubbling for 5 min	80 mW power, 3 µs dwell time, 0.5 µm step size, 20× objective	NaCl (0.02 M for 15 min) → $MgCl_2$ (0.1, 0.3, and 0.5 M for 30 min each) → $CaCl_2$ (0.1, 0.3,	None	None

				and 0.5 M for 30 min each)		
5e, 5f, S12	MSF hydrogel	250 µM rhodamine B, 10 mM H_2O_2, isopropylamine added to tune pH to ~ 8.0-9.5, O_2 bubbling for 5 min	100 mW power, 3 µs dwell time, 0.5 µm step size, 20× objective	NaCl (0.02 M for 15 min) → $MgCl_2$ (0.1, 0.3, and 0.5 M for 30 min each) → $CaCl_2$ (0.1, 0.3, and 0.5 M for 30 min each)	Yes	Yes
6a	MSF hydrogel	250 µM rhodamine B, 10 mM H_2O_2, isopropylamine added to tune pH to ~ 8.0-9.5, O_2 bubbling for 5 min	80 mW power, 3 µs dwell time, 0.5 µm step size, 20× objective	NaCl (0.02 M for 15 min) → $MgCl_2$ (0.1, 0.3, and 0.5 M for 30 min each) → $CaCl_2$ (0.1, 0.3, and 0.5 M for 30 min each)	Yes	Yes
S1b	MSF hydrogel	250 µM rhodamine B, 10 mM H_2O_2, isopropylamine added to tune pH to ~ 8.0-9.5, O_2 bubbling for 5 min	2-109 mW power, 0.79 µs dwell time, 2.0 µm step size, 40× objective	NaCl (0.02 M for 15 min) → $MgCl_2$ (0.1, 0.3, and 0.5 M for 30 min each)	None	None
S1c	MSF hydrogel	250 µM rhodamine B, isopropylamine added to tune pH to ~ 8.0-9.5, O_2 bubbling for 5 min	2-109 mW power, 0.79 µs dwell time, 2.0 µm step size, 40× objective	NaCl (0.02 M for 15 min) → $MgCl_2$ (0.1, 0.3, and 0.5 M for 30 min each)	None	None
S1d	MSF hydrogel	250 µM rhodamine B, isopropylamine added to tune pH to ~ 8.0-9.5, N_2 bubbling for 5 min	2-109 mW power, 0.79 µs dwell time, 2.0 µm step size, 40× objective	NaCl (0.02 M for 15 min) → $MgCl_2$ (0.1, 0.3, and 0.5 M for 30 min each)	None	None
S6g	Agarose hydrogel	1 mM rhodamine B, 50 mM H_2O_2, isopropylamine added to tune pH to ~ 8.0-9.5, O_2 bubbling for 5 min	200 mW power, 6 µs dwell time, 1.0 µm step size, 20× objective	PBS (1×) (no noticeable shrinkage)	None	None
S6h	Alginate hydrogel	1 mM rhodamine B, 50 mM H_2O_2, isopropylamine added to tune pH to ~ 8.0-9.5, O_2 bubbling for 5 min	200 mW power, 6 µs dwell time, 1.0 µm step size, 20× objective	PBS (1×) (modest shrinkage)	None	None
S6i	Gelatin hydrogel	1 mM rhodamine B, 50 mM H_2O_2, isopropylamine added to tune pH to ~ 8.0-9.5, O_2 bubbling for 5 min	200 mW power, 6 µs dwell time, 1.0 µm step size, 20× objective	PBS (1×) (no noticeable shrinkage)	None	None
S7d	MSF hydrogel	250 µM methylene blue, 10 mM H_2O_2, isopropylamine added to tune pH to ~ 8.0-9.5, O_2 bubbling for 5 min	2-109 mW power, 0.79 µs dwell time, 2.0 µm step size, 40× objective	NaCl (0.02 M for 15 min) → $MgCl_2$ (0.1, 0.3, and 0.5 M for 30 min each)	None	None
S7e	MSF hydrogel	250 µM rhodamine 123, 10 mM H_2O_2, isopropylamine added to tune pH to ~ 8.0-9.5, O_2 bubbling for 5 min	2-109 mW power, 0.79 µs dwell time, 2.0 µm step size, 40× objective	NaCl (0.02 M for 15 min) → $MgCl_2$ (0.1, 0.3, and 0.5 M for 30 min each)	None	None

S8	HSF hydrogel	150 µM rhodamine B	60-150 mW power, 1-2 µs dwell time, 2.0 µm step size, 20× objective	NaCl (0.02 M for 15 min) → $MgCl_2$ (0.1, 0.3, and 0.5 M for 30 min each)	None	None
S9	MSF hydrogel	None	None	NaCl (0.02 M for 15 min) → $MgCl_2$ (0.1, 0.3, and 0.5 M for 30 min each) → $CaCl_2$ (0.1, 0.3, and 0.5 M for 30 min each)	Yes	Yes
S10	MSF hydrogel	250 µM rhodamine B, 10 mM H_2O_2, isopropylamine added to tune pH to ~ 8.0-9.5, O_2 bubbling for 5 min	60-100 mW power, 4 µs dwell time, 0.2 µm step size, 20× objective	NaCl (0.02 M for 15 min) → $MgCl_2$ (0.1, 0.3, and 0.5 M for 30 min each) → $CaCl_2$ (0.1, 0.3, and 0.5 M for 30 min each)	Yes	Yes
S11	HSF hydrogel	150 µM rhodamine B, 10 mM H_2O_2, isopropylamine added to tune pH to ~ 8.0-9.5, O_2 bubbling for 5 min	30 mW power, 6 µs dwell time, 0.5 µm step size, 20× objective	NaCl (0.02 M for 15 min) → $MgCl_2$ (0.1, 0.3, and 0.5 M for 30 min each) → $CaCl_2$ (0.1, 0.3, and 0.5 M for 30 min each)	Yes	Yes
S16	HSF hydrogel	150 µM rhodamine B, 10 mM H_2O_2, isopropylamine added to tune pH to ~ 8.0-9.5, O_2 bubbling for 5 min	30 mW power, 6 µs dwell time, 1.7 µm step size, 20× objective	NaCl (0.02 M for 15 min) → $MgCl_2$ (0.1, 0.3, and 0.5 M for 30 min each)	None	None
S17	MSF hydrogel	250 µM rhodamine B, 10 mM H_2O_2, isopropylamine added to tune pH to ~ 8.0-9.5, O_2 bubbling for 5 min	100 mW power, 3 µs dwell time, 1.0 µm step size, 20× objective	NaCl (0.02 M for 15 min) → $MgCl_2$ (0.1, 0.3, and 0.5 M for 30 min each) → $CaCl_2$ (0.1, 0.3, and 0.5 M for 30 min each)	None	None

References

1. Lin, X. *et al.* All-optical machine learning using diffractive deep neural networks. *Science (1979).* **361**, 1004–1008 (2018).
2. Rahman, M. S. S., Yang, X., Li, J., Bai, B. & Ozcan, A. Universal linear intensity transformations using spatially incoherent diffractive processors. *Light Sci. Appl.* **12**, 195 (2023).
3. Goodman, J. W. *Introduction to Fourier Optics*. (Roberts & Co., Greenwood Village, 2005).
4. Mengu, D. & Ozcan, A. All-optical phase recovery: diffractive computing for quantitative phase imaging. *Adv. Opt. Mater.* **10**, (2022).
5. Paschotta, R. Effective refractive index - an encyclopedia article. in *RP Photonics Encyclopedia* (RP Photonics AG, 2006). doi:10.61835/avr.
6. Freund, R. J., Wilson, W. J. & Mohr, D. L. Multiple regression. in *Statistical Methods* 375–471 (Elsevier, 2010). doi:10.1016/B978-0-12-374970-3.00008-1.
7. Saha, S. K. *et al.* Scalable submicrometer additive manufacturing. *Science (1979).* **366**, 105–109 (2019).
8. DeForest, C. A. & Anseth, K. S. Cytocompatible click-based hydrogels with dynamically tunable properties through orthogonal photoconjugation and photocleavage reactions. *Nat. Chem.* **3**, 925–931 (2011).
9. Tibbitt, M. W., Kloxin, A. M., Dyamenahalli, K. U. & Anseth, K. S. Controlled two-photon photodegradation of PEG hydrogels to study and manipulate subcellular interactions on soft materials. *Soft Matter* **6**, 5100 (2010).
10. Batalov, I. *et al.* Grayscale 4D biomaterial customization at high resolution and scale. *bioRxiv* (2024).
11. Rapp, T. L. & DeForest, C. A. Tricolor visible wavelength-selective photodegradable hydrogel biomaterials. *Nat. Commun.* **14**, 5250 (2023).
12. Hiraoka, K., Shin, H. & Yokoyama, T. Density measurements of poly(acrylic acid) sodium salts. *Polymer Bulletin* **8**, (1982).
